# Supplementary material for: CMGL: confidence-guided multi-omics graph learning for cancer subtype classification
Source: Bioinformatics. 2026 Oct 12;42(10):btag763. doi: 10.1093/bioinformatics/btag763 (PMC13712188; doi:10.1093/bioinformatics/btag763)
Supplement: Supplementary file 1 [file btag763_supplementary_data.pdf]

# Supplementary Contents

**A. Pseudocode of the CMGL Procedures**
Algorithm S1. CMGL two-stage training ..... 2
Algorithm S2. Consistency intersection graph construction ..... 2

**B. Supplementary Results for the Main Model**
Table S1. Mathematical notation ..... 3
Table S2. BH-adjusted paired comparisons ..... 5
Figure S1. Main benchmark bar chart ..... 4
Figure S2. Pan-cancer radar chart ..... 4
Table S3. Small-sample generalization ..... 5
Table S4. Full ablation results ..... 6
Table S5. Graph-construction comparison ..... 6
Table S6. Modality-count summary ..... 6
Table S7. Complete modality combinations ..... 7
Table S8. Modality removal results ..... 7
Table S9. Seed-specific confidence results ..... 8
Table S10. Hyperparameter sensitivity grid ..... 8
**C. Supplementary Biological Interpretability Analyses**
Figure S3. GO profiles for BRCA Classes 0, 1, and 3 ..... 9
Figure S4. BRCA embedding visualization ..... 10
Figure S5. BRCA marker-gene heatmap ..... 10
Table S11. Extended BRCA pathway enrichment ..... 11
Table S12. KIRC cluster overview ..... 11
Table S13. KIRC survival summaries ..... 12
Table S14. Pairwise log-rank tests ..... 12
Table S15. KIRC marker genes ..... 12
Figure S6. KIRC GO profiles at $K = 2$ ..... 11
Figure S7. KIRC GO profiles at $K = 3$ ..... 13
Figure S8. KIRC GO profiles at $K = 4$ ..... 13

## A. Pseudocode of the CMGL Procedures

Algorithm S1 formalizes the two-stage CMGL training procedure: Stage 1 estimates per-sample, per-omics confidence through evidential deep learning combined with a quality estimator, and Stage 2 performs confidence-guided fusion and graph-based message passing under frozen confidence priors. Algorithm S2 details the consistency intersection graph construction that supplies the edge index used in Stage 2 and prevents a single omics modality from independently introducing patient adjacencies.

**Algorithm S1** CMGL two-stage training

**Require:** Multi-omics features $\{\mathbf{X}^{(m)}\}_{m=1}^{M}$, labels $\mathbf{y}$, class count $C$, current task, seed, and outer fold, candidate set $\mathcal{K}$, epochs $E_1, E_2$
**Ensure:** Trained $\Theta_r$ and $\Theta_g$, fold-specific $k^*$, frozen confidence $\mathbf{R}$, held-out predictions $\hat{\mathbf{p}}_{\mathrm{te}}$

Stratify the current outer fold into $\mathcal{D}_{\mathrm{tr}}$, $\mathcal{D}_{\mathrm{val}}$, and $\mathcal{D}_{\mathrm{te}}$ (approximately 70%/10%/20%)
**// Stage 1: evidence-driven omics confidence learning**
**for** epoch $= 1, \ldots, E_1$ on $\mathcal{D}_{\mathrm{tr}}$ **do**
    **for** $m = 1, \ldots, M$ **do**
        $\mathbf{s}^{(m)} \leftarrow E_m^{(r)}(\mathbf{X}^{(m)})$;   $\mathbf{e}^{(m)} \leftarrow \mathrm{Softplus}(\mathrm{MLP}(\mathbf{s}^{(m)}))$
        $\boldsymbol{\alpha}^{(m)} \leftarrow \mathbf{e}^{(m)} + \mathbf{1}_C$;   $S^{(m)} \leftarrow \sum_c \alpha_c^{(m)}$;   $\boldsymbol{\pi}^{(m)} \leftarrow \boldsymbol{\alpha}^{(m)}/S^{(m)}$;   $u^{(m)} \leftarrow C/S^{(m)}$
        $q^{(m)} \leftarrow Q_m\left(\log(1 + \sum_c e_c^{(m)}), u^{(m)}, \widetilde{H}^{(m)}, \max_c \pi_c^{(m)}\right)$
    **end for**
    $\mathbf{R} \leftarrow \mathrm{Softmax}([q^{(1)}, \ldots, q^{(M)}]/T)$
    $\Theta_r \leftarrow \mathrm{AdamUpdate}(\Theta_r, \nabla\mathcal{L}_{\mathrm{stage1}})$
**end for**
Freeze $\mathbf{R}$ for all samples
**for** $k \in \mathcal{K}$ **do**
    Build train and validation graphs with Algorithm S2; fit a short Stage 2 candidate and record validation Macro-F1 $v_k$
**end for**
$k^* \leftarrow \arg\max_{k\in\mathcal{K}} v_k$ for the current task, seed, and outer fold; resolve ties in favor of the larger $k$
**// Stage 2: confidence-guided fusion and graph reasoning**
Construct the final train and validation graphs at $k^*$ using Algorithm S2
**for** epoch $= 1, \ldots, E_2$ **do**
    **for** $m = 1, \ldots, M$ **do**
        $\mathbf{v}^{(m)} \leftarrow E_m^{(f)}(\mathbf{X}^{(m)}) + \mathbf{o}_m$
    **end for**
    $\widetilde{\mathbf{V}} \leftarrow \mathrm{MultiHeadAttn}(\mathbf{V}, \mathbf{V}, \mathbf{V})$
    $\mathbf{g}^{(m)} \leftarrow \sigma(\mathbf{W}_g[\widetilde{\mathbf{v}}^{(m)} \| r^{(m)}] + \mathbf{b}_g)$;   $\mathbf{z} \leftarrow \sum_m r^{(m)}(\mathbf{g}^{(m)} \odot \widetilde{\mathbf{v}}^{(m)})$
    $\mathbf{h} \leftarrow \mathrm{GraphSAGE}_{\text{2-layer}}(\mathbf{z}, \mathcal{E}_\cap) + \mathbf{W}_{\mathrm{res}}\mathbf{z}$;   $\hat{\mathbf{p}} \leftarrow \mathrm{Softmax}(\mathbf{W}_{\mathrm{cls}}\mathbf{h} + \mathbf{b}_{\mathrm{cls}})$
    $\Theta_g \leftarrow \mathrm{AdamUpdate}(\Theta_g, \nabla\mathcal{L}_{\mathrm{stage2}})$; retain the checkpoint selected by validation Macro-F1
**end for**
Apply the fold-specific $k^*$ when constructing the train–test graph and obtain predictions for the corresponding outer-fold evaluation
**return** $\Theta_r, \Theta_g, k^*, \mathbf{R}, \hat{\mathbf{p}}_{\mathrm{te}}$

**Algorithm S2** Consistency intersection graph construction

**Require:** Training nodes $\mathcal{V}_{\mathrm{tr}}$, evaluation nodes $\mathcal{V}_{\mathrm{ev}}$, multi-omics features $\{\mathbf{X}^{(m)}\}_{m=1}^{M}$, neighbor count $k$
**Ensure:** Train-only graph $\mathcal{E}_{\cap,\mathrm{tr}}$ and train–evaluation graph $\mathcal{E}_{\cap,\mathrm{tr,ev}}$

**for** $m = 1, \ldots, M$ **do**
    Standardize training and evaluation features using the training-split statistics and compute cosine distances
    $\mathcal{E}_{\mathrm{tr}}^{(m)} \leftarrow \mathrm{Sym}\{(i,j) : i, j \in \mathcal{V}_{\mathrm{tr}},\ j \in \mathrm{NN}_k(\mathbf{x}_i^{(m)})\}$
    $\mathcal{E}_{\mathrm{tr,ev}}^{(m)} \leftarrow \mathrm{Sym}(\mathcal{E}_{\mathrm{tr}}^{(m)} \cup \{$train–evaluation $k$-NN pairs$\})$   ▷ no evaluation–evaluation edges
**end for**
$\mathcal{E}_{\cap,\mathrm{tr}} \leftarrow (\bigcap_m \mathcal{E}_{\mathrm{tr}}^{(m)}) \cup \{(i,i) : i \in \mathcal{V}_{\mathrm{tr}}\}$
$\mathcal{E}_{\cap,\mathrm{tr,ev}} \leftarrow (\bigcap_m \mathcal{E}_{\mathrm{tr,ev}}^{(m)}) \cup \{(i,i) : i \in \mathcal{V}_{\mathrm{tr}} \cup \mathcal{V}_{\mathrm{ev}}\}$
**return** $\mathcal{E}_{\cap,\mathrm{tr}}, \mathcal{E}_{\cap,\mathrm{tr,ev}}$

## B. Supplementary Results for the Main Model

The tables and figures in this section provide the complete numerical evidence underlying the benchmark, robustness, ablation, graph-construction, modality-availability, confidence, and hyperparameter analyses summarized in the main text. They retain the matched task, seed, fold, validation, and training settings used for the corresponding comparisons, while Table S1 supplies a consolidated reference for the notation used throughout the CMGL formulation.

**Table S1** Mathematical notation used in the CMGL method. Symbols are grouped by method component and defined individually in the Definition column.

| Symbol | Definition |
|---|---|
| **Data, indices, and splits** | |
| $i, j, \ell, m, m', c;\ N, M, C$ | $i$ : patient index; $j$ : patient index used for a neighbor or contrastive comparison; $\ell$ : denominator index in the contrastive loss; $m$ : omics-modality index; $m'$ : second modality index used in normalization; $c$ : class index; $N$ : number of patients; $M$ : number of omics modalities; $C$ : number of classes. |
| $\mathcal{D}, \mathbf{x}_i^{(m)}, y_i$ | $\mathcal{D}$ : complete patient cohort; $\mathbf{x}_i^{(m)}$ : input vector for patient $i$ in modality $m$; $y_i$ : class label of patient $i$. |
| $\mathbf{X}^{(m)}, \mathbf{y};\ \mathcal{D}_{\mathrm{tr/val/te}}, \mathcal{V}_{\mathrm{tr/val/te}}$ | $\mathbf{X}^{(m)}$ : data matrix for modality $m$; $\mathbf{y}$ : vector of patient class labels; $\mathcal{D}_{\mathrm{tr}}$ : training split; $\mathcal{D}_{\mathrm{val}}$ : validation split; $\mathcal{D}_{\mathrm{te}}$ : held-out test split; $\mathcal{V}_{\mathrm{tr}}$ : training-node set; $\mathcal{V}_{\mathrm{val}}$ : validation-node set; $\mathcal{V}_{\mathrm{te}}$ : test-node set. |
| $\Theta_r, \Theta_g$ | $\Theta_r$ : trainable parameters of the Stage 1 confidence model; $\Theta_g$ : trainable parameters of the Stage 2 fusion and graph-classification model. |
| **Stage 1: Evidence Modeling** | |
| $\mathbf{e}_i^{(m)}, \boldsymbol{\alpha}_i^{(m)}, S_i^{(m)}, \mathbf{1}_C$ | $\mathbf{e}_i^{(m)}$ : non-negative class-wise evidence; $\boldsymbol{\alpha}_i^{(m)}$ : Dirichlet parameter vector; $S_i^{(m)}$ : total Dirichlet strength; $\mathbf{1}_C$ : length-$C$ all-ones vector. |
| $\boldsymbol{\pi}_i^{(m)}, u_i^{(m)}$ | $\boldsymbol{\pi}_i^{(m)}$ : Dirichlet predictive-mean vector; $u_i^{(m)}$ : epistemic uncertainty. |
| **Stage 1: Quality Estimator** | |
| $a_i^{(m)}, \widetilde{H}_i^{(m)}, \pi_{i,\max}^{(m)}$ | $a_i^{(m)}$ : log evidence strength; $\widetilde{H}_i^{(m)}$ : normalized predictive entropy; $\pi_{i,\max}^{(m)}$ : maximum predictive probability. |
| $Q_m, T, r_i^{(m)}, \mathbf{r}_i, \mathbf{R}$ | $Q_m$ : modality-specific quality-scoring network; $T$ : learned positive temperature; $r_i^{(m)}$ : scalar confidence for modality $m$ and patient $i$; $\mathbf{r}_i$ : confidence vector for patient $i$; $\mathbf{R}$ : confidence matrix for all patients and modalities. |
| **Stage 2: Cross-omics Attention and Fusion** | |
| $E_m^{(r)}, E_m^{(f)}, \mathbf{o}_m$ | $E_m^{(r)}$ : evidence encoder for modality $m$; $E_m^{(f)}$ : fusion encoder for modality $m$; $\mathbf{o}_m$ : learnable identity vector for modality $m$. |
| $\mathbf{v}_i^{(m)}, \widetilde{\mathbf{v}}_i^{(m)}$ | $\mathbf{v}_i^{(m)}$ : pre-attention modality token; $\widetilde{\mathbf{v}}_i^{(m)}$ : attended modality token. |
| $\mathbf{g}_i^{(m)}, \mathbf{z}_i, \mathbf{Z}$ | $\mathbf{g}_i^{(m)}$ : feature-wise gating vector; $\mathbf{z}_i$ : confidence-guided fused representation; $\mathbf{Z}$ : stacked node-feature matrix. |
| **Consistency Intersection Graph Construction** | |
| $k, \mathcal{K}, k^*, \mathcal{E}^{(m)}, \mathcal{E}_\cap$ | $k$ : nearest-neighbor count; $\mathcal{K}$ : candidate set of neighbor counts; $k^*$ : validation-selected neighbor count; $\mathcal{E}^{(m)}$ : modality-$m$ $k$-NN edge set; $\mathcal{E}_\cap$ : intersection of modality-specific edge sets. |
| **Graph Message Passing and Classification** | |
| $\mathbf{h}_i, \widetilde{\mathbf{h}}_i, \hat{\mathbf{p}}_i$ | $\mathbf{h}_i$ : residual GraphSAGE embedding of patient $i$; $\widetilde{\mathbf{h}}_i$ : normalized residual embedding; $\hat{\mathbf{p}}_i$ : predicted class-probability vector. |
| **Stage 1 and Stage 2 Losses** | |
| $\mathcal{B}, \mathcal{B}_+, \mathcal{P}(i), \widetilde{y}_{i,c}, \mathbb{I}[\cdot]$ | $\mathcal{B}$ : training-node set used for Stage 2 losses; $\mathcal{B}_+$ : training anchors with at least one positive example; $\mathcal{P}(i)$ : same-class positive set for anchor $i$; $\widetilde{y}_{i,c}$ : label-smoothed target for class $c$; $\mathbb{I}[\cdot]$ : indicator function. |
| $w_{y_i}, \epsilon, \tau$ | $w_{y_i}$ : class weight for patient $i$; $\epsilon$ : label-smoothing coefficient; $\tau$ : supervised-contrastive temperature. |
| $\mathcal{L}_{\mathrm{edl}}, \mathcal{L}_{\mathrm{cls}}^{(r)}, \mathcal{L}_{\mathrm{div}}, \mathcal{L}_{\mathrm{ce}}, \mathcal{L}_{\mathrm{supcon}}, \mathcal{L}_{\mathrm{stage1}}, \mathcal{L}_{\mathrm{stage2}}$ | $\mathcal{L}_{\mathrm{edl}}$ : evidential deep-learning loss; $\mathcal{L}_{\mathrm{cls}}^{(r)}$ : confidence-weighted Stage 1 classification loss; $\mathcal{L}_{\mathrm{div}}$ : confidence-diversity regularization loss; $\mathcal{L}_{\mathrm{ce}}$ : label-smoothing cross-entropy loss; $\mathcal{L}_{\mathrm{supcon}}$ : supervised contrastive loss; $\mathcal{L}_{\mathrm{stage1}}$ : total Stage 1 loss; $\mathcal{L}_{\mathrm{stage2}}$ : total Stage 2 loss. |
| $\lambda_{\mathrm{edl}}, \lambda_{\mathrm{cls}}^{(r)}, \lambda_{\mathrm{div}}, \lambda_{\mathrm{cls}}, \lambda_{\mathrm{con}}$ | $\lambda_{\mathrm{edl}}$ : coefficient of the evidential loss; $\lambda_{\mathrm{cls}}^{(r)}$ : coefficient of the confidence-weighted classification loss; $\lambda_{\mathrm{div}}$ : coefficient of the diversity loss; $\lambda_{\mathrm{cls}}$ : coefficient of the Stage 2 classification loss; $\lambda_{\mathrm{con}}$ : coefficient of the contrastive loss. |
| **Parameters and operators** | |
| $\mathbf{W}_g, \mathbf{b}_g, \mathbf{W}_{\mathrm{res}}, \mathbf{W}_{\mathrm{cls}}, \mathbf{b}_{\mathrm{cls}}$ | $\mathbf{W}_g$ : feature-gate weight matrix; $\mathbf{b}_g$ : feature-gate bias vector; $\mathbf{W}_{\mathrm{res}}$ : residual-projection matrix; $\mathbf{W}_{\mathrm{cls}}$ : classifier weight matrix; $\mathbf{b}_{\mathrm{cls}}$ : classifier bias vector. |
| $[\cdot\Vert\cdot], \odot, \sigma, \Vert\cdot\Vert_2$ | $[\cdot\Vert\cdot]$ : vector concatenation; $\odot$ : elementwise multiplication; $\sigma$ : sigmoid activation function; $\Vert\cdot\Vert_2$ : Euclidean norm. |

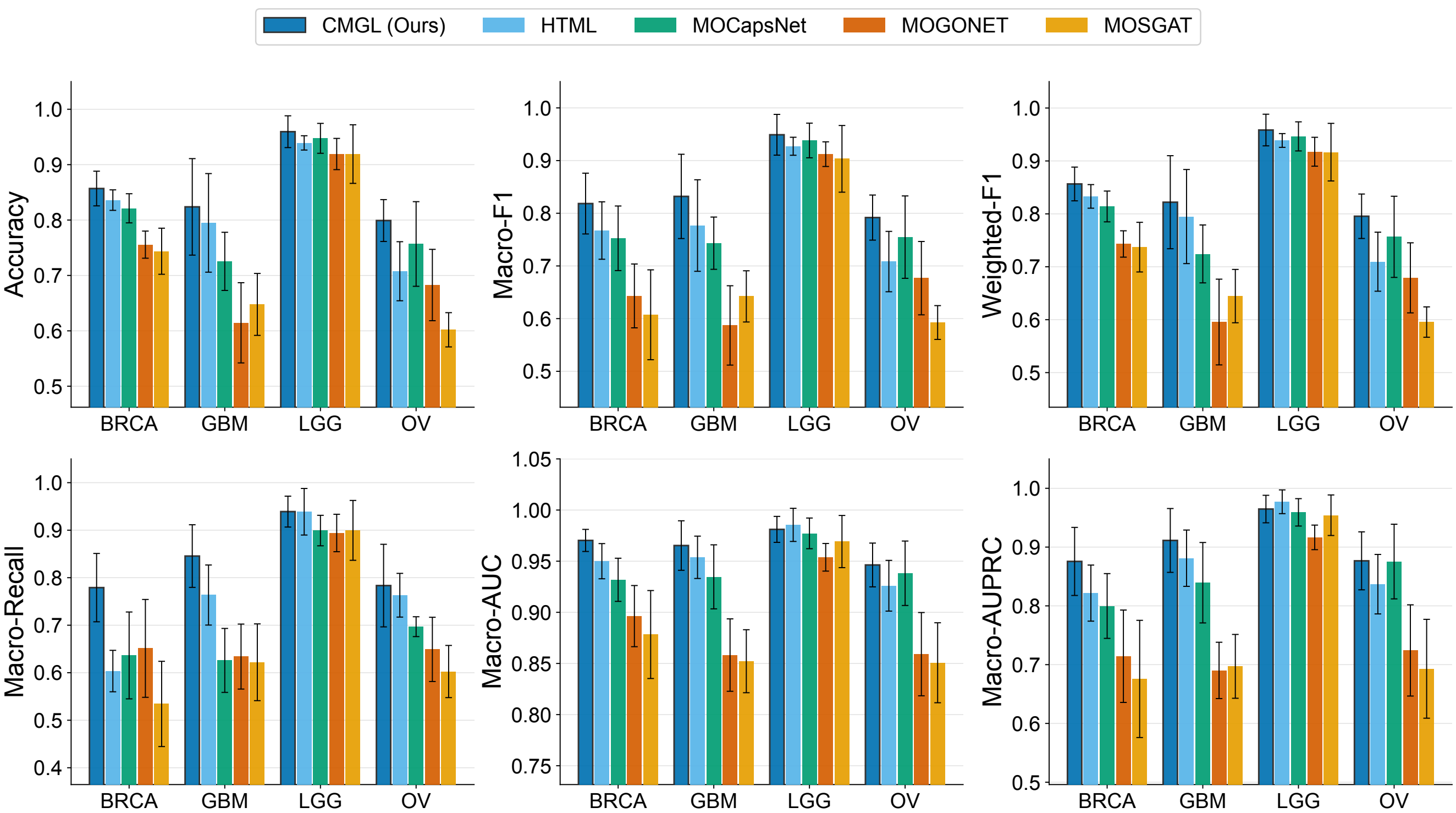


**Figure S1** Grouped bar charts of six benchmark metrics across five methods and four cancer subtype tasks under 5-fold cross-validation; error bars indicate standard deviation.

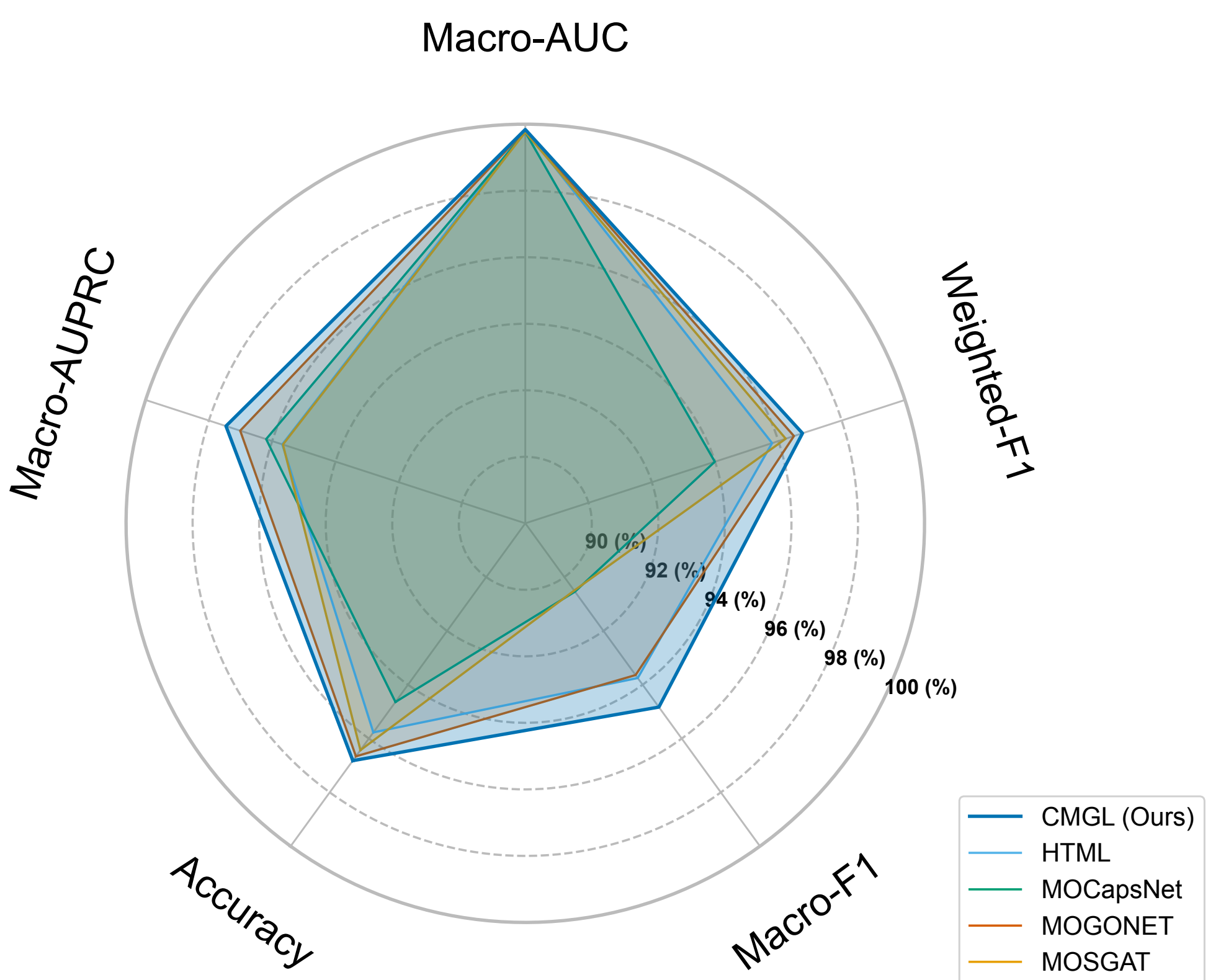


**Figure S2** Radar-chart visualization of five displayed pan-cancer metrics from Table 3 of the main paper under 5-fold cross-validation.

**Table S2** Paired tests between CMGL and the baselines across 15 evaluations from three seeds and five folds. Each metric reports $\Delta = \text{baseline} - \text{CMGL}$ and the two-sided Wilcoxon $p_{\text{adj}}$ after correction across 80 comparisons. Negative differences favor CMGL, and asterisks denote $p_{\text{adj}} < 0.05$.

| Task | Baseline | Δ Accuracy | $p_{\text{adj}}$ | Δ Macro-F1 | $p_{\text{adj}}$ | Δ Macro-AUC | $p_{\text{adj}}$ | Δ Macro-AUPRC | $p_{\text{adj}}$ |
|---|---|---|---|---|---|---|---|---|---|
| BRCA | HTML | -0.0611 | 0.0001* | -0.1793 | 0.0001* | -0.0288 | 0.0003* | -0.0711 | 0.0001* |
| | MOGONET | -0.0542 | 0.0014* | -0.1156 | 0.0002* | -0.0579 | 0.0001* | -0.1385 | 0.0001* |
| | MOSGAT | -0.1580 | 0.0001* | -0.2707 | 0.0001* | -0.1026 | 0.0001* | -0.2371 | 0.0001* |
| | MOCapsNet | -0.0561 | 0.0001* | -0.1215 | 0.0001* | -0.0425 | 0.0002* | -0.0940 | 0.0002* |
| GBM | HTML | -0.0492 | 0.0294* | -0.0570 | 0.0091* | -0.0048 | 0.6874 | -0.0175 | 0.3076 |
| | MOGONET | -0.1559 | 0.0003* | -0.1613 | 0.0007* | -0.0928 | 0.0001* | -0.1663 | 0.0002* |
| | MOSGAT | -0.3635 | 0.0001* | -0.3975 | 0.0001* | -0.2586 | 0.0001* | -0.4043 | 0.0001* |
| | MOCapsNet | -0.1229 | 0.0005* | -0.1450 | 0.0009* | -0.0474 | 0.0005* | -0.0973 | 0.0009* |
| LGG | HTML | +0.0244 | 0.0615 | +0.0280 | 0.0420* | +0.0026 | 0.4233 | +0.0088 | 0.0876 |
| | MOGONET | -0.0375 | 0.0763 | -0.0423 | 0.0763 | -0.0310 | 0.0002* | -0.0419 | 0.0007* |
| | MOSGAT | -0.0768 | 0.0005* | -0.0886 | 0.0003* | -0.0380 | 0.0022* | -0.0662 | 0.0013* |
| | MOCapsNet | -0.0404 | 0.0046* | -0.0482 | 0.0102* | -0.0106 | 0.0952 | -0.0175 | 0.1066 |
| OV | HTML | -0.0176 | 0.4657 | -0.0164 | 0.5758 | -0.0050 | 0.5758 | -0.0136 | 0.4846 |
| | MOGONET | -0.1468 | 0.0001* | -0.1510 | 0.0001* | -0.1260 | 0.0001* | -0.2252 | 0.0001* |
| | MOSGAT | -0.3359 | 0.0001* | -0.3496 | 0.0001* | -0.2405 | 0.0001* | -0.3791 | 0.0001* |
| | MOCapsNet | -0.1126 | 0.0002* | -0.1123 | 0.0002* | -0.0572 | 0.0001* | -0.0970 | 0.0001* |
| Pan-cancer | HTML | -0.0131 | 0.0001* | -0.0593 | 0.0001* | +0.0002 | 0.5144 | -0.0169 | 0.0001* |
| | MOGONET | +0.0001 | 0.9341 | -0.0261 | 0.0002* | +0.0006 | 0.0283* | -0.0074 | 0.0681 |
| | MOSGAT | -0.0027 | 0.0059* | -0.0395 | 0.0001* | -0.0006 | 0.0951 | -0.0208 | 0.0002* |
| | MOCapsNet | -0.0408 | 0.0001* | -0.1662 | 0.0001* | -0.0301 | 0.0001* | -0.1338 | 0.0001* |

**Table S3** Small-sample generalization across the four single-cancer tasks under 5-fold cross-validation (mean ± std), providing the numerical results underlying Fig. 2 of the main paper. CMGL provides the strongest overall performance, especially on BRCA and GBM, while the bold entries identify the few LGG and OV settings led by HTML or MOCapsNet. Bold indicates the best result per task and training fraction.

| Task / Frac. | Model | Accuracy | Macro-F1 | Macro-AUC | Macro-AUPRC |
|---|---|---|---|---|---|
| BRCA 0.3 | CMGL | **0.8092 ± 0.0310** | **0.7107 ± 0.0581** | **0.9530 ± 0.0089** | **0.7789 ± 0.0330** |
| | HTML | 0.7794 ± 0.0509 | 0.6340 ± 0.0830 | 0.9325 ± 0.0194 | 0.7472 ± 0.0574 |
| | MOCapsNet | 0.6736 ± 0.0513 | 0.5391 ± 0.0658 | 0.8629 ± 0.0270 | 0.6330 ± 0.0389 |
| | MOGONET | 0.7302 ± 0.0494 | 0.5369 ± 0.1005 | 0.8586 ± 0.0502 | 0.6027 ± 0.1006 |
| | MOSGAT | 0.5604 ± 0.0641 | 0.2849 ± 0.0898 | 0.6688 ± 0.0850 | 0.3731 ± 0.0687 |
| BRCA 0.5 | CMGL | **0.8271 ± 0.0278** | **0.7422 ± 0.0540** | **0.9585 ± 0.0133** | **0.8384 ± 0.0487** |
| | HTML | 0.8048 ± 0.0227 | 0.7113 ± 0.0277 | 0.9355 ± 0.0179 | 0.7761 ± 0.0429 |
| | MOCapsNet | 0.7824 ± 0.0193 | 0.6746 ± 0.0359 | 0.9040 ± 0.0211 | 0.7264 ± 0.0361 |
| | MOGONET | 0.7555 ± 0.0361 | 0.5970 ± 0.0786 | 0.8961 ± 0.0209 | 0.6548 ± 0.0743 |
| | MOSGAT | 0.6795 ± 0.0292 | 0.3963 ± 0.0552 | 0.7854 ± 0.0392 | 0.5063 ± 0.0428 |
| GBM 0.3 | CMGL | **0.6930 ± 0.0903** | **0.6703 ± 0.0786** | 0.9146 ± 0.0414 | 0.7933 ± 0.0763 |
| | HTML | 0.6886 ± 0.0672 | 0.6748 ± 0.0757 | **0.9252 ± 0.0270** | **0.8073 ± 0.0617** |
| | MOCapsNet | 0.5656 ± 0.0637 | 0.5771 ± 0.0593 | 0.8485 ± 0.0412 | 0.6678 ± 0.0739 |
| | MOGONET | 0.5944 ± 0.0998 | 0.5950 ± 0.1219 | 0.8213 ± 0.0523 | 0.6523 ± 0.0918 |
| | MOSGAT | 0.3565 ± 0.0469 | 0.3120 ± 0.0793 | 0.6301 ± 0.0716 | 0.4017 ± 0.0767 |
| GBM 0.5 | CMGL | **0.7912 ± 0.0836** | **0.7856 ± 0.0881** | **0.9552 ± 0.0280** | **0.8854 ± 0.0777** |
| | HTML | 0.7173 ± 0.0741 | 0.6886 ± 0.0767 | 0.9267 ± 0.0414 | 0.8088 ± 0.0995 |
| | MOCapsNet | 0.6762 ± 0.0398 | 0.6949 ± 0.0347 | 0.9083 ± 0.0309 | 0.7878 ± 0.0525 |
| | MOGONET | 0.6148 ± 0.0550 | 0.6076 ± 0.0432 | 0.8005 ± 0.0388 | 0.6585 ± 0.0618 |
| | MOSGAT | 0.4761 ± 0.1085 | 0.4042 ± 0.1510 | 0.6682 ± 0.0875 | 0.4733 ± 0.1323 |
| LGG 0.3 | CMGL | **0.9229 ± 0.0328** | 0.9102 ± 0.0401 | 0.9707 ± 0.0176 | 0.9513 ± 0.0344 |
| | HTML | 0.9230 ± 0.0239 | **0.9130 ± 0.0357** | **0.9764 ± 0.0212** | **0.9653 ± 0.0309** |
| | MOCapsNet | 0.9068 ± 0.0703 | 0.8822 ± 0.0936 | 0.9628 ± 0.0260 | 0.9411 ± 0.0459 |
| | MOGONET | 0.8989 ± 0.0462 | 0.8855 ± 0.0533 | 0.9417 ± 0.0391 | 0.9140 ± 0.0461 |
| | MOSGAT | 0.7653 ± 0.0785 | 0.7205 ± 0.1451 | 0.8419 ± 0.0837 | 0.7637 ± 0.1097 |
| LGG 0.5 | CMGL | 0.9312 ± 0.0162 | 0.9163 ± 0.0277 | 0.9745 ± 0.0161 | 0.9575 ± 0.0295 |
| | HTML | **0.9433 ± 0.0154** | **0.9322 ± 0.0224** | **0.9850 ± 0.0186** | **0.9766 ± 0.0237** |
| | MOCapsNet | 0.8744 ± 0.0485 | 0.8512 ± 0.0563 | 0.9639 ± 0.0174 | 0.9411 ± 0.0256 |
| | MOGONET | 0.8950 ± 0.0338 | 0.8833 ± 0.0411 | 0.9468 ± 0.0239 | 0.9077 ± 0.0336 |
| | MOSGAT | 0.8257 ± 0.0464 | 0.8024 ± 0.0348 | 0.9241 ± 0.0398 | 0.8601 ± 0.0733 |
| OV 0.3 | CMGL | 0.6443 ± 0.0266 | 0.6297 ± 0.0419 | 0.8581 ± 0.0269 | 0.7195 ± 0.0567 |
| | HTML | 0.6055 ± 0.0759 | 0.6075 ± 0.0782 | **0.9013 ± 0.0372** | **0.7904 ± 0.0767** |
| | MOCapsNet | **0.6475 ± 0.0727** | **0.6499 ± 0.0714** | 0.8722 ± 0.0447 | 0.7709 ± 0.0709 |
| | MOGONET | 0.5877 ± 0.0457 | 0.5792 ± 0.0415 | 0.7960 ± 0.0610 | 0.6423 ± 0.0934 |
| | MOSGAT | 0.3284 ± 0.1456 | 0.2772 ± 0.1376 | 0.6333 ± 0.1217 | 0.4292 ± 0.1464 |
| OV 0.5 | CMGL | **0.7181 ± 0.0401** | **0.7120 ± 0.0382** | 0.8941 ± 0.0239 | 0.7768 ± 0.0272 |
| | HTML | 0.5452 ± 0.1101 | 0.5434 ± 0.1169 | 0.8881 ± 0.0502 | 0.7896 ± 0.0785 |
| | MOCapsNet | 0.7071 ± 0.1074 | 0.7077 ± 0.1058 | **0.9081 ± 0.0451** | **0.8227 ± 0.0783** |
| | MOGONET | 0.6685 ± 0.0877 | 0.6611 ± 0.0924 | 0.8549 ± 0.0485 | 0.7092 ± 0.0792 |
| | MOSGAT | 0.4753 ± 0.0479 | 0.4237 ± 0.0639 | 0.7433 ± 0.0485 | 0.5360 ± 0.0444 |

**Table S4** Full numerical results corresponding to the ablation heatmap in Fig. 3 of the main paper under 5-fold cross-validation. Each row removes one core module from CMGL; cross-omics attention fusion has the largest average effect, with its removal reducing Accuracy by 3.0% and Macro-F1 by 4.1%. Bold indicates the best result in each column.

| | Accuracy | | | | |
|---|---|---|---|---|---|
| Variant | BRCA | GBM | LGG | OV | Average |
| CMGL (Full) | **0.857** | **0.824** | **0.960** | **0.799** | **0.860** |
| w/o Uncertainty-Aware | 0.842 | 0.766 | 0.946 | 0.790 | 0.836 |
| w/o Cross-Omics Fusion | 0.851 | 0.816 | 0.954 | 0.700 | 0.830 |
| w/o Two-Stage Framework | 0.848 | 0.779 | 0.955 | 0.792 | 0.843 |
| | Macro-F1 | | | | |
| Variant | BRCA | GBM | LGG | OV | Average |
| CMGL (Full) | **0.818** | **0.832** | **0.949** | **0.792** | **0.848** |
| w/o Uncertainty-Aware | 0.780 | 0.782 | 0.934 | 0.782 | 0.820 |
| w/o Cross-Omics Fusion | 0.796 | 0.798 | 0.944 | 0.691 | 0.807 |
| w/o Two-Stage Framework | 0.804 | 0.791 | 0.945 | 0.787 | 0.832 |
| | Macro-AUC | | | | |
| Variant | BRCA | GBM | LGG | OV | Average |
| CMGL (Full) | **0.970** | 0.965 | 0.981 | **0.946** | **0.966** |
| w/o Uncertainty-Aware | 0.967 | 0.942 | 0.974 | 0.937 | 0.955 |
| w/o Cross-Omics Fusion | 0.963 | **0.966** | 0.974 | 0.906 | 0.952 |
| w/o Two-Stage Framework | 0.964 | 0.950 | **0.982** | **0.946** | 0.961 |

**Table S5** Matched comparison of graph strategies on the four datasets. Performance values and density are mean ± SD across three seeds. Density measures the undirected graph without self loops.

| Task | Strategy | Accuracy | Macro-F1 | Macro-AUC | Macro-AUPRC | Density |
|---|---|---|---|---|---|---|
| BRCA | Intersection | $0.8495 \pm 0.0093$ | $0.8000 \pm 0.0185$ | $0.9673 \pm 0.0024$ | $0.8682 \pm 0.0171$ | $0.0031 \pm 0.0004$ |
| | Union | $0.8455 \pm 0.0134$ | $0.7853 \pm 0.0368$ | $0.9661 \pm 0.0054$ | $0.8617 \pm 0.0310$ | $0.1792 \pm 0.0192$ |
| | Confidence-topk | $0.8435 \pm 0.0186$ | $0.7888 \pm 0.0333$ | $0.9646 \pm 0.0039$ | $0.8559 \pm 0.0166$ | $0.0305 \pm 0.0048$ |
| GBM | Intersection | $0.7993 \pm 0.0674$ | $0.8043 \pm 0.0634$ | $0.9497 \pm 0.0222$ | $0.8815 \pm 0.0533$ | $0.0224 \pm 0.0025$ |
| | Union | $0.8061 \pm 0.0450$ | $0.8150 \pm 0.0389$ | $0.9545 \pm 0.0183$ | $0.8896 \pm 0.0343$ | $0.5120 \pm 0.0036$ |
| | Confidence-topk | $0.7939 \pm 0.0663$ | $0.7948 \pm 0.0644$ | $0.9480 \pm 0.0294$ | $0.8825 \pm 0.0564$ | $0.1082 \pm 0.0035$ |
| LGG | Intersection | $0.9406 \pm 0.0123$ | $0.9287 \pm 0.0160$ | $0.9818 \pm 0.0051$ | $0.9669 \pm 0.0084$ | $0.0291 \pm 0.0067$ |
| | Union | $0.9392 \pm 0.0143$ | $0.9274 \pm 0.0125$ | $0.9795 \pm 0.0069$ | $0.9651 \pm 0.0076$ | $0.5267 \pm 0.0462$ |
| | Confidence-topk | $0.9337 \pm 0.0084$ | $0.9188 \pm 0.0111$ | $0.9805 \pm 0.0042$ | $0.9643 \pm 0.0073$ | $0.1188 \pm 0.0156$ |
| OV | Intersection | $0.8040 \pm 0.0600$ | $0.7989 \pm 0.0595$ | $0.9545 \pm 0.0212$ | $0.8966 \pm 0.0481$ | $0.0225 \pm 0.0021$ |
| | Union | $0.8075 \pm 0.0511$ | $0.8015 \pm 0.0495$ | $0.9517 \pm 0.0204$ | $0.8932 \pm 0.0407$ | $0.4408 \pm 0.0225$ |
| | Confidence-topk | $0.8039 \pm 0.0328$ | $0.7967 \pm 0.0284$ | $0.9546 \pm 0.0141$ | $0.8960 \pm 0.0299$ | $0.0943 \pm 0.0062$ |

**Table S6** Average performance under cohort-level modality-subset retraining. Each subset model is initialized and trained using only the available views; their modality-specific $k$-NN graphs are intersected before confidence-guided fusion and GraphSAGE classification under the matched split and validation-selected $k$. Two-view and three-view columns average all six and four combinations, respectively; Full-4 is the matched reference.

| Task | Metric | Avg. two-view | Avg. three-view | Full-4 |
|---|---|---|---|---|
| BRCA | Accuracy | 0.8145 | 0.8338 | 0.8495 |
| | Macro-F1 | 0.7441 | 0.7759 | 0.8000 |
| GBM | Accuracy | 0.7136 | 0.7456 | 0.7993 |
| | Macro-F1 | 0.7081 | 0.7448 | 0.8043 |
| LGG | Accuracy | 0.8943 | 0.9336 | 0.9406 |
| | Macro-F1 | 0.8749 | 0.9229 | 0.9287 |
| OV | Accuracy | 0.7299 | 0.7471 | 0.8040 |
| | Macro-F1 | 0.7234 | 0.7405 | 0.7989 |

**Table S7** Modality-combination retraining results. Each subset is trained using only its available views; the corresponding $k$-NN graph intersection supports confidence-guided fusion and GraphSAGE classification under the matched protocol. All two-view, three-view, and Full-4-view settings are shown.

| Task | Setting | Accuracy | Macro-F1 | Macro-AUC | Macro-AUPRC |
|---|---|---|---|---|---|
| BRCA | Full-4-view [ref] | 0.8495 ± 0.0093 | 0.8000 ± 0.0185 | 0.9673 ± 0.0024 | 0.8682 ± 0.0171 |
| | mRNA+miRNA | 0.8510 ± 0.0039 | 0.7997 ± 0.0083 | 0.9674 ± 0.0040 | 0.8694 ± 0.0103 |
| | mRNA+Methy | 0.8564 ± 0.0090 | 0.8132 ± 0.0309 | 0.9697 ± 0.0054 | 0.8740 ± 0.0170 |
| | mRNA+CNV | 0.8356 ± 0.0126 | 0.7738 ± 0.0254 | 0.9609 ± 0.0057 | 0.8572 ± 0.0314 |
| | miRNA+Methy | 0.8053 ± 0.0009 | 0.7310 ± 0.0190 | 0.9467 ± 0.0066 | 0.8055 ± 0.0056 |
| | miRNA+CNV | 0.7740 ± 0.0220 | 0.6766 ± 0.0340 | 0.9378 ± 0.0090 | 0.7585 ± 0.0325 |
| | Methy+CNV | 0.7645 ± 0.0186 | 0.6706 ± 0.0411 | 0.9308 ± 0.0033 | 0.7558 ± 0.0202 |
| | mRNA+miRNA+Methy | 0.8505 ± 0.0087 | 0.7993 ± 0.0116 | 0.9687 ± 0.0019 | 0.8682 ± 0.0140 |
| | mRNA+miRNA+CNV | 0.8490 ± 0.0130 | 0.8044 ± 0.0156 | 0.9676 ± 0.0067 | 0.8750 ± 0.0111 |
| | mRNA+Methy+CNV | 0.8326 ± 0.0167 | 0.7652 ± 0.0286 | 0.9595 ± 0.0102 | 0.8417 ± 0.0307 |
| | miRNA+Methy+CNV | 0.8033 ± 0.0161 | 0.7346 ± 0.0313 | 0.9474 ± 0.0003 | 0.7985 ± 0.0080 |
| GBM | Full-4-view [ref] | 0.7993 ± 0.0674 | 0.8043 ± 0.0634 | 0.9497 ± 0.0222 | 0.8815 ± 0.0533 |
| | mRNA+miRNA | 0.8130 ± 0.0144 | 0.8014 ± 0.0125 | 0.9608 ± 0.0070 | 0.8878 ± 0.0130 |
| | mRNA+Methy | 0.8158 ± 0.0497 | 0.8204 ± 0.0479 | 0.9536 ± 0.0184 | 0.8922 ± 0.0325 |
| | mRNA+CNV | 0.7953 ± 0.0567 | 0.7947 ± 0.0586 | 0.9543 ± 0.0215 | 0.8816 ± 0.0530 |
| | miRNA+Methy | 0.6830 ± 0.0048 | 0.6806 ± 0.0135 | 0.8908 ± 0.0090 | 0.7701 ± 0.0114 |
| | miRNA+CNV | 0.5615 ± 0.0854 | 0.5436 ± 0.0796 | 0.8348 ± 0.0403 | 0.6475 ± 0.0678 |
| | Methy+CNV | 0.6132 ± 0.0358 | 0.6078 ± 0.0559 | 0.8531 ± 0.0211 | 0.6950 ± 0.0280 |
| | mRNA+miRNA+Methy | 0.8007 ± 0.0473 | 0.8019 ± 0.0428 | 0.9501 ± 0.0172 | 0.8805 ± 0.0330 |
| | mRNA+miRNA+CNV | 0.7637 ± 0.0684 | 0.7537 ± 0.0677 | 0.9449 ± 0.0305 | 0.8651 ± 0.0649 |
| | mRNA+Methy+CNV | 0.7966 ± 0.0684 | 0.7994 ± 0.0676 | 0.9444 ± 0.0320 | 0.8724 ± 0.0622 |
| | miRNA+Methy+CNV | 0.6215 ± 0.0405 | 0.6241 ± 0.0318 | 0.8647 ± 0.0232 | 0.7071 ± 0.0502 |
| LGG | Full-4-view [ref] | 0.9406 ± 0.0123 | 0.9287 ± 0.0160 | 0.9818 ± 0.0051 | 0.9669 ± 0.0084 |
| | mRNA+miRNA | 0.9298 ± 0.0091 | 0.9167 ± 0.0110 | 0.9819 ± 0.0096 | 0.9678 ± 0.0127 |
| | mRNA+Methy | 0.9162 ± 0.0386 | 0.8992 ± 0.0460 | 0.9611 ± 0.0195 | 0.9368 ± 0.0294 |
| | mRNA+CNV | 0.9393 ± 0.0042 | 0.9287 ± 0.0040 | 0.9809 ± 0.0025 | 0.9659 ± 0.0035 |
| | miRNA+Methy | 0.7180 ± 0.0500 | 0.6721 ± 0.0570 | 0.8732 ± 0.0374 | 0.7852 ± 0.0465 |
| | miRNA+CNV | 0.9312 ± 0.0039 | 0.9174 ± 0.0067 | 0.9717 ± 0.0098 | 0.9516 ± 0.0155 |
| | Methy+CNV | 0.9313 ± 0.0106 | 0.9150 ± 0.0156 | 0.9678 ± 0.0018 | 0.9479 ± 0.0013 |
| | mRNA+miRNA+Methy | 0.9192 ± 0.0137 | 0.9065 ± 0.0175 | 0.9747 ± 0.0122 | 0.9567 ± 0.0189 |
| | mRNA+miRNA+CNV | 0.9353 ± 0.0201 | 0.9274 ± 0.0234 | 0.9808 ± 0.0035 | 0.9660 ± 0.0026 |
| | mRNA+Methy+CNV | 0.9446 ± 0.0086 | 0.9326 ± 0.0083 | 0.9795 ± 0.0029 | 0.9650 ± 0.0062 |
| | miRNA+Methy+CNV | 0.9352 ± 0.0290 | 0.9251 ± 0.0318 | 0.9830 ± 0.0072 | 0.9693 ± 0.0111 |
| OV | Full-4-view [ref] | 0.8040 ± 0.0600 | 0.7989 ± 0.0595 | 0.9545 ± 0.0212 | 0.8966 ± 0.0481 |
| | mRNA+miRNA | 0.8521 ± 0.0231 | 0.8468 ± 0.0213 | 0.9697 ± 0.0106 | 0.9295 ± 0.0230 |
| | mRNA+Methy | 0.8604 ± 0.0467 | 0.8555 ± 0.0462 | 0.9721 ± 0.0113 | 0.9369 ± 0.0230 |
| | mRNA+CNV | 0.8264 ± 0.0661 | 0.8193 ± 0.0630 | 0.9548 ± 0.0273 | 0.9003 ± 0.0568 |
| | miRNA+Methy | 0.6807 ± 0.0214 | 0.6735 ± 0.0219 | 0.8823 ± 0.0081 | 0.7664 ± 0.0180 |
| | miRNA+CNV | 0.5493 ± 0.0615 | 0.5431 ± 0.0652 | 0.7965 ± 0.0499 | 0.6220 ± 0.0705 |
| | Methy+CNV | 0.6103 ± 0.0663 | 0.6023 ± 0.0718 | 0.8416 ± 0.0495 | 0.6969 ± 0.0777 |
| | mRNA+miRNA+Methy | 0.8088 ± 0.0500 | 0.8016 ± 0.0512 | 0.9618 ± 0.0123 | 0.9101 ± 0.0235 |
| | mRNA+miRNA+CNV | 0.7947 ± 0.0709 | 0.7895 ± 0.0694 | 0.9417 ± 0.0351 | 0.8675 ± 0.0648 |
| | mRNA+Methy+CNV | 0.7630 ± 0.0814 | 0.7553 ± 0.0831 | 0.9293 ± 0.0466 | 0.8512 ± 0.0809 |
| | miRNA+Methy+CNV | 0.6220 ± 0.0403 | 0.6157 ± 0.0438 | 0.8537 ± 0.0372 | 0.7072 ± 0.0582 |

**Table S8** Modality level confidence and classification changes after modality removal over 15 matched evaluations from three seeds and five outer folds. Mean confidence, $\Delta p_{\mathrm{true}}$, and $\Delta$NLL are mean ± SD; Accuracy and Macro-F1 are held-out scores after removing each modality, with changes from the matched Full-4 result in parentheses.

**B. Modality-level confidence and classification changes**

| Task | Removed modality | Confidence | $\Delta p_{\mathrm{true}}$ | $\Delta$NLL | Accuracy ($\Delta$) | Macro-F1 ($\Delta$) |
|---|---|---|---|---|---|---|
| BRCA | mRNA | $0.6424 \pm 0.4681$ | $0.2015 \pm 0.1234$ | $0.3401 \pm 0.2051$ | 0.7113 (-0.1382) | 0.6587 (-0.1413) |
| | miRNA | $0.0101 \pm 0.0049$ | $0.0041 \pm 0.0033$ | $0.0048 \pm 0.0088$ | 0.8460 (-0.0035) | 0.7966 (-0.0034) |
| | Methy | $0.2518 \pm 0.3603$ | $0.0166 \pm 0.0261$ | $0.0480 \pm 0.0691$ | 0.8331 (-0.0164) | 0.7796 (-0.0204) |
| | CNV | $0.0958 \pm 0.1581$ | $-0.0020 \pm 0.0045$ | $-0.0030 \pm 0.0196$ | 0.8445 (-0.0050) | 0.7839 (-0.0161) |
| GBM | mRNA | $0.6482 \pm 0.4288$ | $0.2093 \pm 0.1277$ | $0.4336 \pm 0.2048$ | 0.6407 (-0.1586) | 0.6407 (-0.1636) |
| | miRNA | $0.0173 \pm 0.0107$ | $-0.0017 \pm 0.0059$ | $-0.0058 \pm 0.0194$ | 0.7993 (+0.0000) | 0.8042 (-0.0001) |
| | Methy | $0.3205 \pm 0.4447$ | $0.0122 \pm 0.0487$ | $-0.0305 \pm 0.1496$ | 0.7951 (-0.0042) | 0.7828 (-0.0215) |
| | CNV | $0.0140 \pm 0.0074$ | $-0.0020 \pm 0.0102$ | $-0.0089 \pm 0.0344$ | 0.7883 (-0.0110) | 0.7956 (-0.0087) |
| LGG | mRNA | $0.3872 \pm 0.4059$ | $0.0830 \pm 0.1123$ | $0.1219 \pm 0.1587$ | 0.9136 (-0.0270) | 0.8951 (-0.0336) |
| | miRNA | $0.0113 \pm 0.0061$ | $-0.0017 \pm 0.0033$ | $-0.0001 \pm 0.0063$ | 0.9405 (-0.0001) | 0.9265 (-0.0022) |
| | Methy | $0.0175 \pm 0.0125$ | $-0.0056 \pm 0.0051$ | $-0.0064 \pm 0.0101$ | 0.9419 (+0.0013) | 0.9299 (+0.0012) |
| | CNV | $0.5840 \pm 0.4222$ | $0.0850 \pm 0.0754$ | $0.1375 \pm 0.1134$ | 0.8893 (-0.0513) | 0.8781 (-0.0506) |
| OV | mRNA | $0.3343 \pm 0.4345$ | $0.1820 \pm 0.1434$ | $0.4121 \pm 0.1985$ | 0.6478 (-0.1562) | 0.6440 (-0.1549) |
| | miRNA | $0.0332 \pm 0.0202$ | $-0.0084 \pm 0.0048$ | $-0.0150 \pm 0.0310$ | 0.8099 (+0.0059) | 0.8049 (+0.0060) |
| | Methy | $0.6243 \pm 0.4519$ | $0.0154 \pm 0.0622$ | $-0.0266 \pm 0.1120$ | 0.8135 (+0.0095) | 0.8068 (+0.0079) |
| | CNV | $0.0082 \pm 0.0045$ | $-0.0206 \pm 0.0110$ | $-0.0560 \pm 0.0410$ | 0.8286 (+0.0246) | 0.8238 (+0.0249) |

**Table S9** Seed-specific modality-confidence results underlying Table 4 in the main paper. Each entry is the mean across five outer folds for seeds 42, 43, and 44, respectively.

| Task | $\rho$(conf., $\Delta p_{\mathrm{true}}$), seeds 42 / 43 / 44 | $\rho$(conf., $\Delta$NLL), seeds 42 / 43 / 44 | Top-1 agreement, seeds 42 / 43 / 44 |
|---|---|---|---|
| BRCA | 0.3901 / 0.4172 / -0.0157 | 0.3815 / 0.4056 / -0.0149 | 0.8480 / 0.8331 / 0.4411 |
| GBM | 0.4247 / 0.2126 / 0.4345 | 0.4118 / 0.1808 / 0.4286 | 0.8325 / 0.5163 / 0.8440 |
| LGG | 0.4483 / 0.3748 / 0.5337 | 0.4322 / 0.3776 / 0.5338 | 0.7452 / 0.8985 / 0.8540 |
| OV | 0.0175 / 0.2677 / 0.4234 | -0.0307 / 0.2411 / 0.4135 | 0.6127 / 0.4088 / 0.8944 |

**Table S10** Full joint hyperparameter sensitivity grid underlying Fig. 4 of the main paper on BRCA under 5-fold cross-validation (mean $\pm$ std). The sub-grids vary ($\lambda_{\mathrm{edl}}$, anneal) for Stage 1 and ($\lambda_{\mathrm{cls}}, \lambda_{\mathrm{con}}$) for Stage 2. Across the 18 configurations, Macro-F1 remains within a $\pm 0.025$ band and Accuracy within $\pm 0.012$, placing the selected operating point $(1.5, 50, 3.0, 1.0)$ within a flat region of the evaluated grid.

| Group | X parameter | Y parameter | Macro-F1 | Accuracy |
|---|---|---|---|---|
| Uncertainty-Aware Learning | $\lambda_{\mathrm{edl}}$=0.5 | anneal= 10 | $0.8254 \pm 0.0556$ | $0.8614 \pm 0.0341$ |
| | $\lambda_{\mathrm{edl}}$=0.5 | anneal= 50 | $0.8314 \pm 0.0556$ | $0.8688 \pm 0.0341$ |
| | $\lambda_{\mathrm{edl}}$=0.5 | anneal= 150 | $0.8254 \pm 0.0556$ | $0.8614 \pm 0.0341$ |
| | $\lambda_{\mathrm{edl}}$=1.5 | anneal= 10 | $0.8254 \pm 0.0556$ | $0.8614 \pm 0.0341$ |
| | $\lambda_{\mathrm{edl}}$=1.5 | anneal= 50 | $0.8314 \pm 0.0556$ | $0.8688 \pm 0.0341$ |
| | $\lambda_{\mathrm{edl}}$=1.5 | anneal= 150 | $0.8314 \pm 0.0556$ | $0.8688 \pm 0.0341$ |
| | $\lambda_{\mathrm{edl}}$=3.0 | anneal= 10 | $0.8314 \pm 0.0556$ | $0.8688 \pm 0.0341$ |
| | $\lambda_{\mathrm{edl}}$=3.0 | anneal= 50 | $0.8254 \pm 0.0556$ | $0.8614 \pm 0.0341$ |
| | $\lambda_{\mathrm{edl}}$=3.0 | anneal= 150 | $0.8314 \pm 0.0556$ | $0.8688 \pm 0.0341$ |
| GNN Loss Balancing | $\lambda_{\mathrm{cls}}$=1.0 | $\lambda_{\mathrm{con}}$=0.5 | $0.8180 \pm 0.0541$ | $0.8599 \pm 0.0319$ |
| | $\lambda_{\mathrm{cls}}$=1.0 | $\lambda_{\mathrm{con}}$=1.0 | $0.7934 \pm 0.0804$ | $0.8524 \pm 0.0326$ |
| | $\lambda_{\mathrm{cls}}$=1.0 | $\lambda_{\mathrm{con}}$=1.5 | $0.7918 \pm 0.0864$ | $0.8584 \pm 0.0277$ |
| | $\lambda_{\mathrm{cls}}$=3.0 | $\lambda_{\mathrm{con}}$=0.5 | $0.8446 \pm 0.0554$ | $0.8718 \pm 0.0323$ |
| | $\lambda_{\mathrm{cls}}$=3.0 | $\lambda_{\mathrm{con}}$=1.0 | $0.8314 \pm 0.0556$ | $0.8688 \pm 0.0341$ |
| | $\lambda_{\mathrm{cls}}$=3.0 | $\lambda_{\mathrm{con}}$=1.5 | $0.8157 \pm 0.0586$ | $0.8599 \pm 0.0346$ |
| | $\lambda_{\mathrm{cls}}$=5.0 | $\lambda_{\mathrm{con}}$=0.5 | $0.8319 \pm 0.0483$ | $0.8688 \pm 0.0295$ |
| | $\lambda_{\mathrm{cls}}$=5.0 | $\lambda_{\mathrm{con}}$=1.0 | $0.8329 \pm 0.0541$ | $0.8688 \pm 0.0283$ |
| | $\lambda_{\mathrm{cls}}$=5.0 | $\lambda_{\mathrm{con}}$=1.5 | $0.8312 \pm 0.0652$ | $0.8688 \pm 0.0381$ |

# C. Supplementary Biological Interpretability Analyses

This section extends the BRCA analyses in Section 3.8 and the BRCA-to-KIRC transfer analyses in Section 3.9 of the main paper. The BRCA results use the frozen-inference outputs of the best model across all 671 samples, while the KIRC results summarize cluster structure, survival, marker genes, and GO enrichment for $K \in \{2, 3, 4\}$, including the selected $K = 3$ solution.

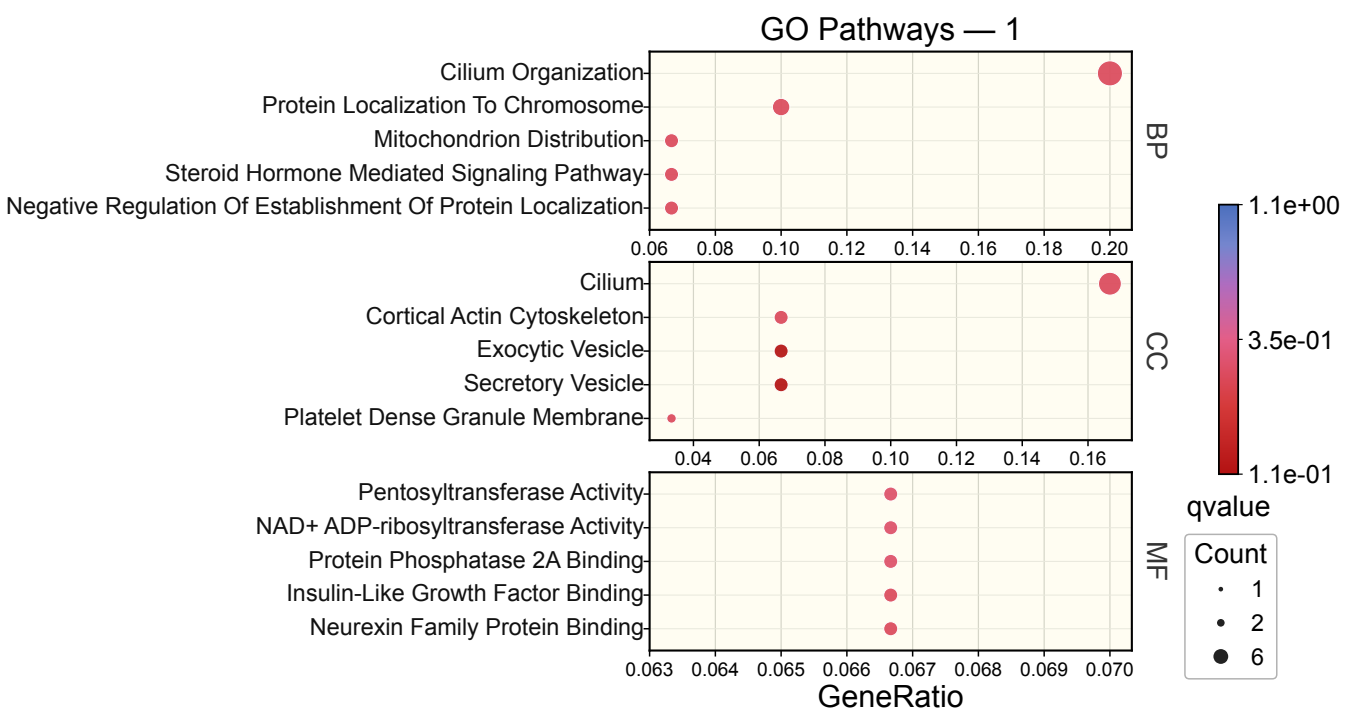


Class 0 (Luminal A)

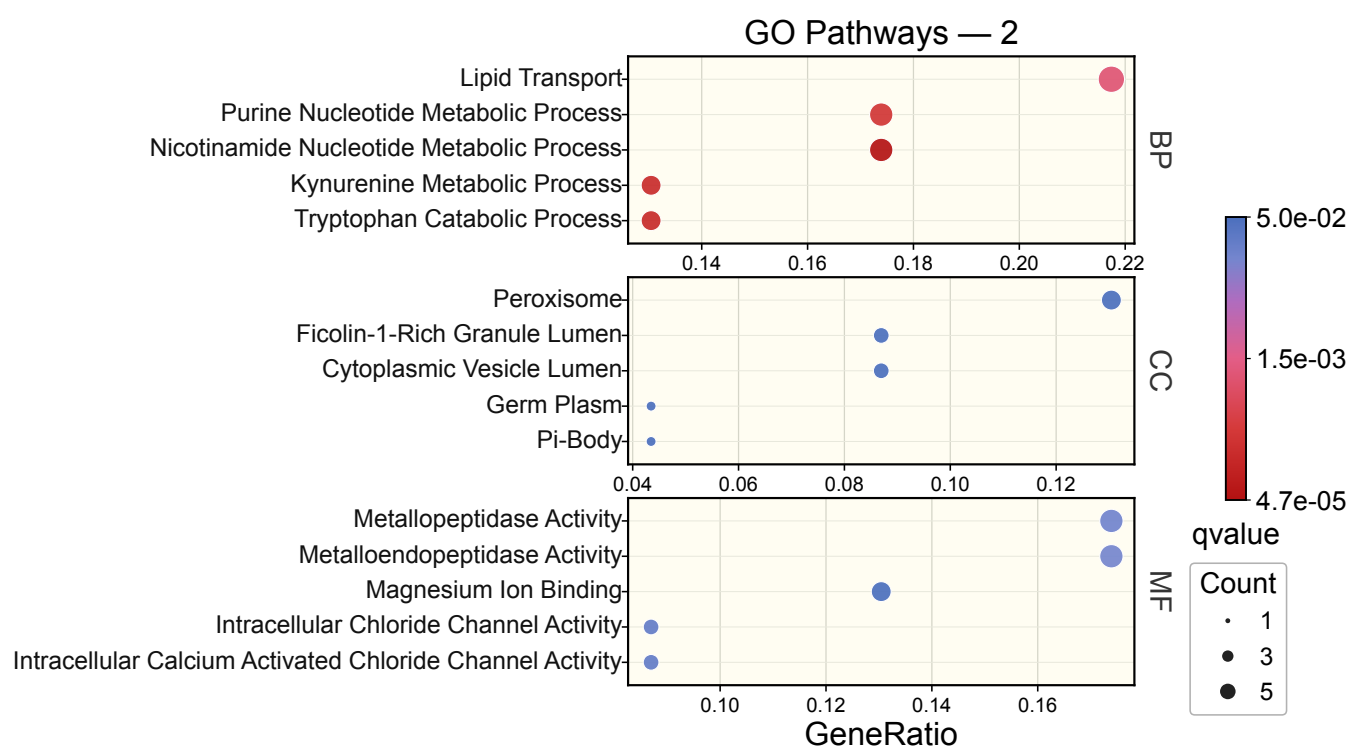


Class 1 (Metabolic)

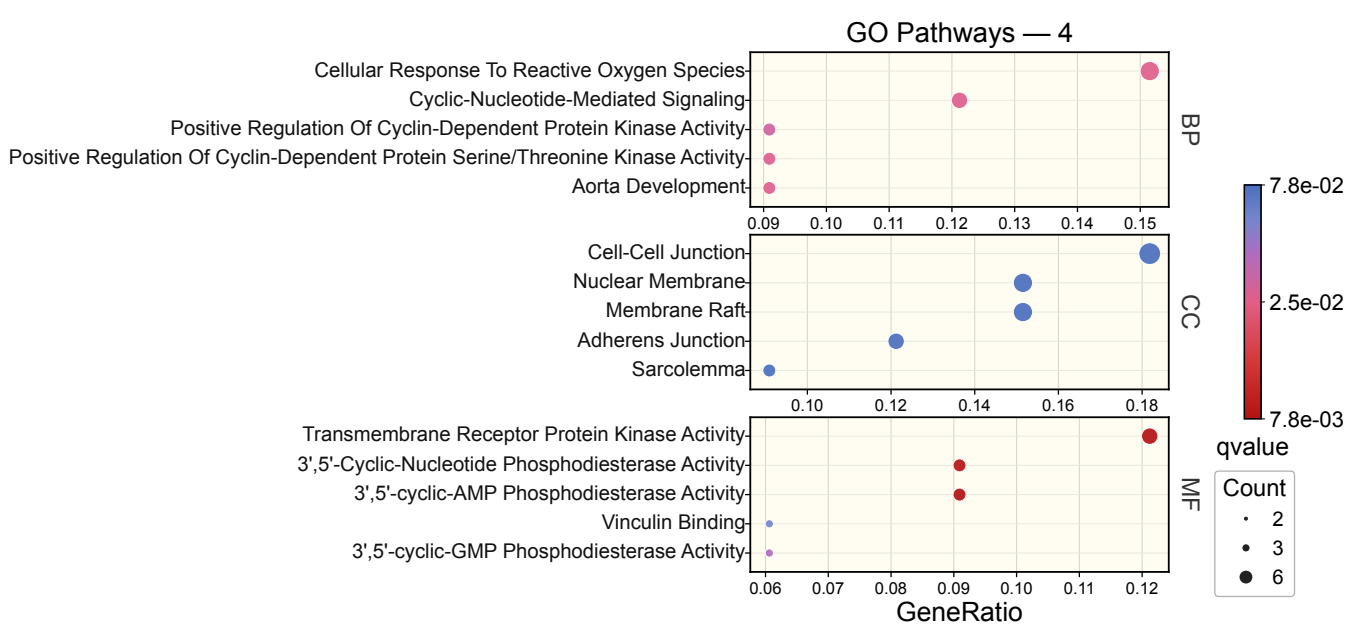


Class 3 (Normal-like)

**Figure S3** GO Biological Process enrichment plots for BRCA Classes 0, 1, and 3, complementing the Class 2 and Class 4 profiles in Fig. 5 of the main paper.

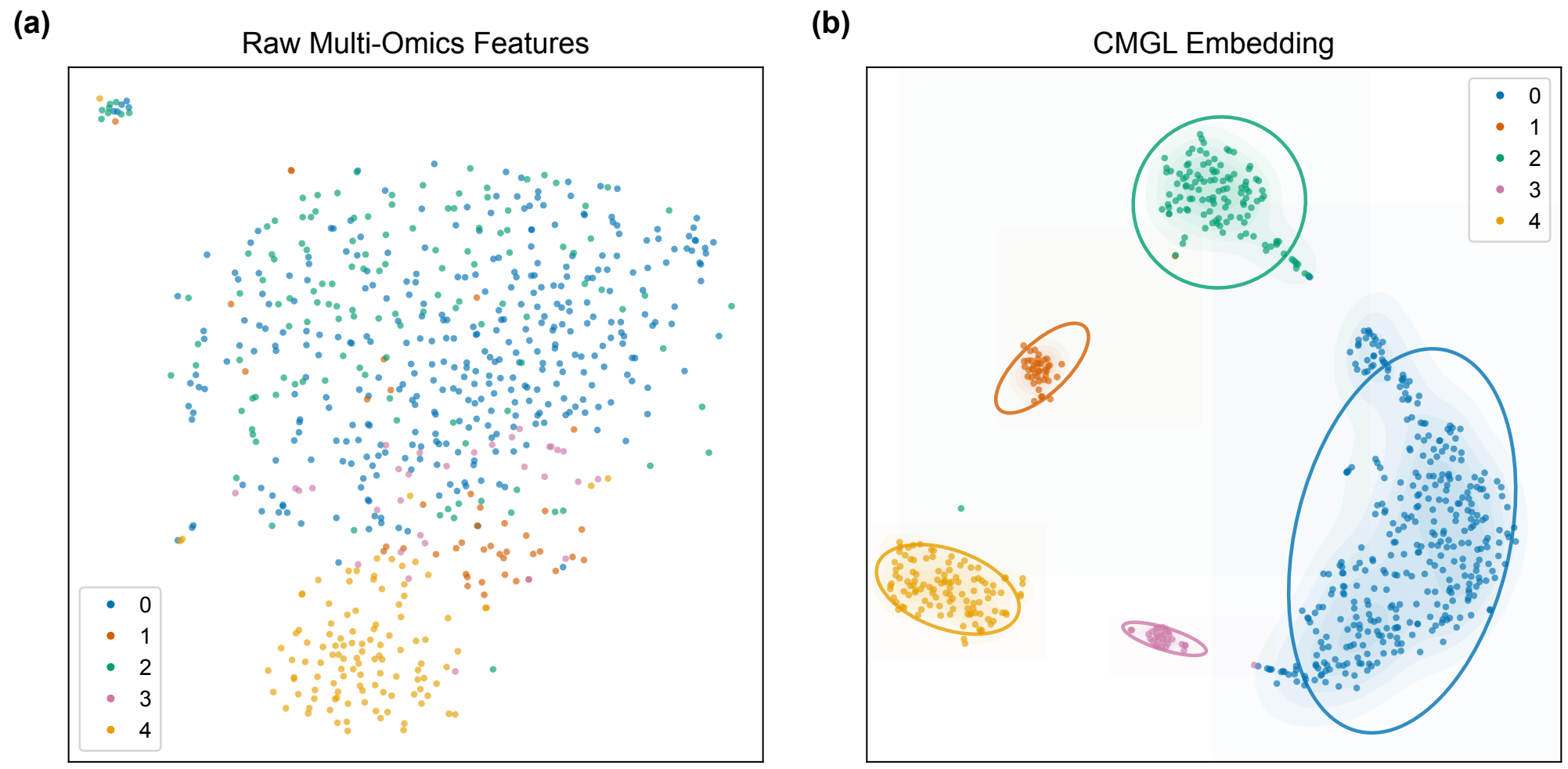


**Figure S4** t-SNE visualization of BRCA subtype embeddings and predicted class structure referenced in Section 3.8 of the main paper.

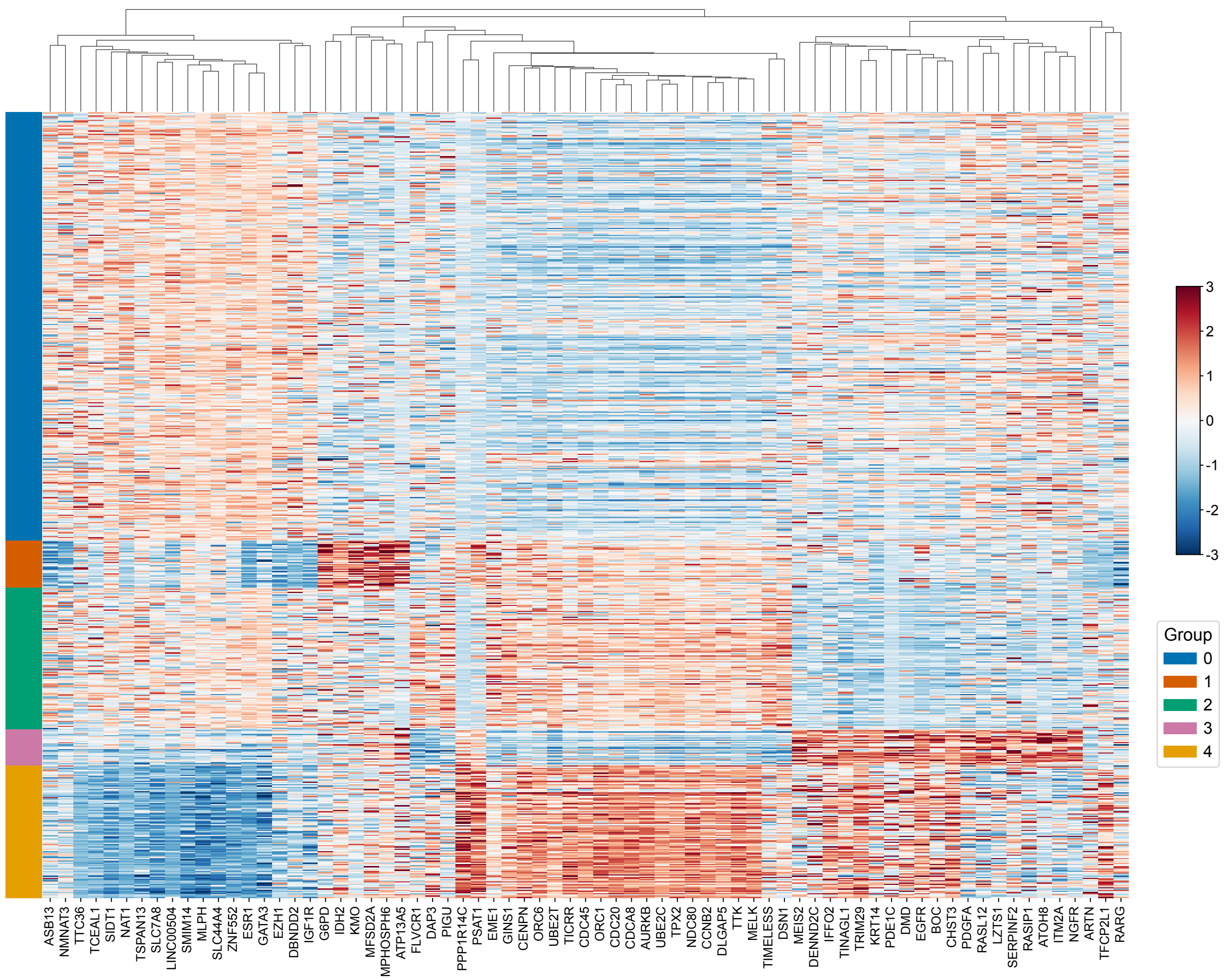


**Figure S5** Heatmap of representative mRNA marker genes across the five predicted BRCA classes, visualizing the transcriptional separation referenced in Section 3.8 of the main paper.

**Table S11** Extended BRCA pathway enrichment listing the top GO Biological Process and KEGG terms for each predicted class and extending Table 5 of the main paper. Class 1 concentrates tryptophan/kynurenine and glutathione metabolism, whereas Classes 2 and 4 share proliferation and cell-cycle signatures. Class 0 has no significant term after correction (leading $p_{\mathrm{adj}} \approx 0.25$) and is instead characterized by down-regulation of mitotic markers under the effect-size criterion. Bold $p$ values indicate $p_{\mathrm{adj}} < 0.05$.

| Class | Library | Top term | Adj. $p$ | Representative genes |
|---|---|---|---|---|
| | GO BP | Steroid Hormone Mediated Signaling | $0.252$ | ESR1, CALCOCO1 |
| 0 | GO BP | Cilium Organization | $0.252$ | DYNC2H1, IFT88, ABLIM3 |
| | KEGG | MAPK signaling pathway | $0.386$ | TGFB3, MAPT, IGF1R, MAP3K12 |
| | GO BP | Nicotinamide Nucleotide Metabolism | $\mathbf{4.7\times10^{-5}}$ | G6PD, IDH1, IDH2, KMO |
| | GO BP | Tryptophan Catabolic Process | $\mathbf{1.3\times10^{-4}}$ | TDO2, KYNU, KMO |
| 1 | GO BP | Kynurenine Metabolic Process | $\mathbf{1.4\times10^{-4}}$ | TDO2, KYNU, KMO |
| | KEGG | Glutathione metabolism | $\mathbf{9.9\times10^{-4}}$ | G6PD, RRM2, IDH1, IDH2 |
| | KEGG | Tryptophan metabolism | $\mathbf{5.6\times10^{-3}}$ | TDO2, KYNU, KMO |
| | GO BP | DNA Metabolic Process | $\mathbf{3.4\times10^{-9}}$ | TOP2A, TYMS, MCM2, RAD51AP1 |
| | GO BP | Mitotic Sister Chromatid Segreg. | $\mathbf{5.1\times10^{-9}}$ | TPX2, KIF14, NUSAP1, NDC80 |
| 2 | GO BP | Microtubule Cyto. Org. in Mitosis | $\mathbf{1.4\times10^{-8}}$ | KIF4A, AURKB, KIF11, NDC80 |
| | KEGG | Cell cycle | $\mathbf{2.1\times10^{-6}}$ | CCNB2, CDC45, PTTG1, MCM2 |
| | KEGG | p53 signaling pathway | $\mathbf{3.4\times10^{-3}}$ | CCNB2, RRM2, CCNE2, GTSE1 |
| | GO BP | Aorta Development | $\mathbf{2.6\times10^{-2}}$ | PDE2A, PKD2, TGFBR2 |
| 3 | GO BP | Pos. Reg. CDK Activity | $\mathbf{2.6\times10^{-2}}$ | CCND2, PKD2, EGFR |
| | KEGG | MAPK signaling pathway | $\mathbf{2.8\times10^{-2}}$ | PDGFRA, EGFR, TGFBR2, NGFR |
| | KEGG | Hippo signaling pathway | $\mathbf{2.8\times10^{-2}}$ | CCND2, SNAI2, TCF7, ID1 |
| | GO BP | Pos. Reg. Sister Chromatid Sep. | $\mathbf{1.6\times10^{-6}}$ | CDC20, UBE2C, CDCA8, AURKB |
| | GO BP | Mitotic Spindle Organization | $\mathbf{1.5\times10^{-5}}$ | GPSM2, TPX2, AURKB, NDC80 |
| 4 | GO BP | Microtubule Cyto. Org. in Mitosis | $\mathbf{2.7\times10^{-5}}$ | GPSM2, CDCA8, TTK, NDC80 |
| | KEGG | Cell cycle | $\mathbf{8.8\times10^{-4}}$ | CDC20, CCNB2, ORC6, ORC1 |
| | KEGG | Cysteine and methionine metabolism | $\mathbf{4.3\times10^{-2}}$ | LDHB, PSAT1, AMD1 |

**Table S12** KIRC cluster overviews across $K \in \{2, 3, 4\}$ under the BRCA-transferred embedding space, including size, dominant source-domain BRCA class, mean confidence, and leading marker for each cluster. The proliferation-driven cluster, dominated by source Class 2, recurs with stable size and high confidence across all resolutions, while source-class membership increases with cluster purity. The selected $K = 3$ solution is discussed in Section 3.9; "Lead displayed marker" is the first marker for the same pair in Table S15.

| $K$ | Cluster | $n$ | Dom. source class (%) | Mean confidence | Lead displayed marker |
|---|---|---|---|---|---|
| 2 | 1 | 101 | Class 2 ($70.3\%$) | $0.749$ | BIRC5 |
| | 2 | 213 | Class 0 ($95.3\%$) | $0.820$ | KAT5 |
| | 1 | 28 | Class 3 ($57.1\%$) | $0.673$ | MARCKSL1 |
| 3 | 2 | 206 | Class 0 ($98.1\%$) | $0.826$ | KAT5 |
| | 3 | 80 | Class 2 ($88.8\%$) | $0.767$ | PPP1R13B |
| | 1 | 197 | Class 0 ($100.0\%$) | $0.842$ | KAT5 |
| 4 | 2 | 16 | Class 4 ($56.3\%$) | $0.599$ | SPOCD1 |
| | 3 | 25 | Class 3 ($68.0\%$) | $0.616$ | JAM3 |
| | 4 | 76 | Class 2 ($93.4\%$) | $0.781$ | PPP1R13B |

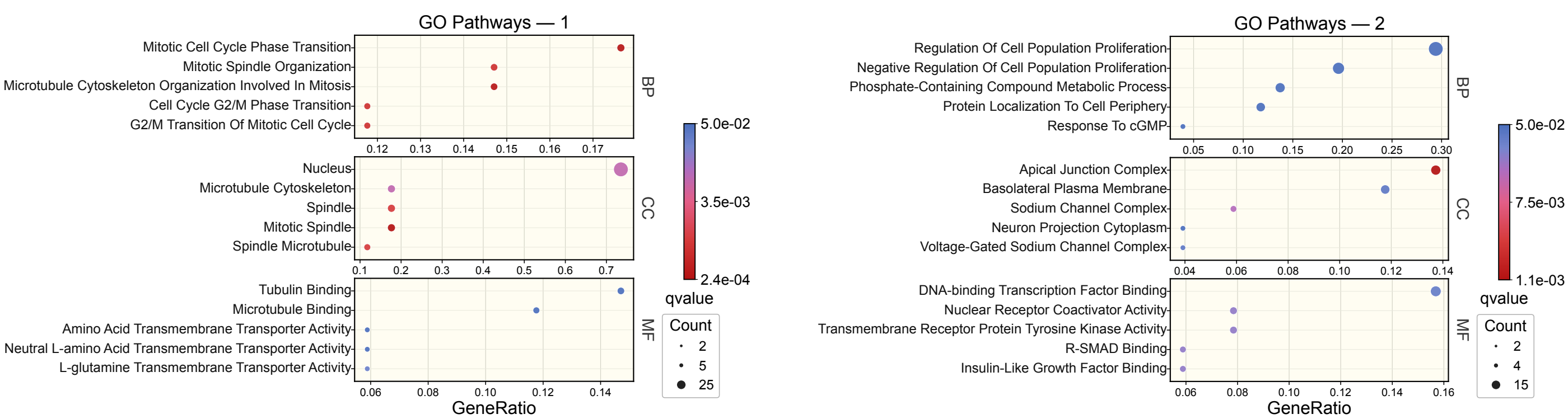


Cluster 1 Cluster 2

**Figure S6** Top GO Biological Process enrichment terms for the two KIRC clusters under $K = 2$.

**Table S13** KIRC DSS and PFI survival analyses across $K = 2, 3, 4$. The proliferation group has a 5-year DSS of $48.9\%$ versus $84.4\%$ and HR $= 4.03$ at $K = 2$; at $K = 3$, the high-risk cluster has HRs of $3.99$ for DSS and $2.74$ for PFI; and at $K = 4$, the two proliferation clusters have DSS HRs of $3.92$ and $3.91$, whereas the mesenchymal cluster is not significant ($p = 0.71$). HRs are relative to the marked reference, and "NR" denotes median survival not reached. Overall log-rank: $K$=2 DSS $p = 6.35\times10^{-10}$, PFI $p = 9.02\times10^{-9}$; $K$=3 DSS $p = 3.66\times10^{-8}$, PFI $p = 1.54\times10^{-6}$; $K$=4 DSS $p = 5.43\times10^{-8}$, PFI $p = 2.11\times10^{-6}$.

| $K$ | Endpt | Cluster | Risk | $n$ | Events (%) | Median (d) | 5-yr Rate | HR (95% CI) | $p$ |
|---|---|---|---|---|---|---|---|---|---|
| 2 | DSS | 2 | low | 206 | 24 (11.7%) | NR | 84.4% | 1.00 (ref) | N/A |
| | | 1 | high | 99 | 42 (42.4%) | 1625 | 48.9% | 4.03 (2.47–6.56) | $2.2\times10^{-8}$ |
| | PFI | 2 | low | 212 | 49 (23.1%) | 3766 | 73.9% | 1.00 (ref) | N/A |
| | | 1 | high | 101 | 54 (53.5%) | 1111 | 33.1% | 2.88 (1.97–4.23) | $6.2\times10^{-8}$ |
| 3 | DSS | 2 | low | 200 | 24 (12.0%) | NR | 83.8% | 1.00 (ref) | N/A |
| | | 1 | int. | 27 | 9 (33.3%) | NR | 66.7% | 2.42 (1.14–5.15) | 0.021 |
| | | 3 | high | 78 | 33 (42.3%) | 1588 | 47.0% | 3.99 (2.39–6.65) | $1.1\times10^{-7}$ |
| | PFI | 2 | low | 205 | 48 (23.4%) | 3766 | 73.5% | 1.00 (ref) | N/A |
| | | 1 | int. | 28 | 14 (50.0%) | 1426 | 43.4% | 2.11 (1.17–3.81) | 0.013 |
| | | 3 | high | 80 | 41 (51.3%) | 1111 | 35.0% | 2.74 (1.81–4.14) | $1.7\times10^{-6}$ |
| 4 | DSS | 1 | low | 191 | 23 (12.0%) | NR | 83.6% | 1.00 (ref) | N/A |
| | | 3 | int. | 24 | 4 (16.7%) | NR | 87.1% | 1.21 (0.43–3.40) | 0.711 |
| | | 2 | high | 15 | 8 (53.3%) | 1371 | 48.5% | 3.92 (1.77–8.68) | $7.7\times10^{-4}$ |
| | | 4 | high | 75 | 31 (41.3%) | 1588 | 46.6% | 3.91 (2.32–6.59) | $3.3\times10^{-7}$ |
| | PFI | 3 | low | 25 | 8 (32.0%) | NR | 66.5% | 1.00 (ref) | N/A |
| | | 1 | int. | 196 | 46 (23.5%) | 3766 | 72.7% | 0.76 (0.39–1.51) | 0.438 |
| | | 2 | high | 16 | 10 (62.5%) | 794 | 38.9% | 2.12 (0.88–5.09) | 0.092 |
| | | 4 | high | 76 | 39 (51.3%) | 951 | 33.0% | 2.16 (1.08–4.32) | 0.029 |

**Table S14** KIRC pairwise log-rank tests across $K \in \{2, 3, 4\}$ for DSS and PFI. At every resolution, the strongest contrast is between the low-risk epithelial/metabolic and high-risk proliferation groups ($K$=2 DSS $p = 6.35\times10^{-10}$; $K$=3 DSS $p = 4.18\times10^{-9}$; $K$=4 Cluster 1 vs. 4 DSS $p = 1.87\times10^{-8}$), with PFI following the same pattern. The mesenchymal/intermediate group separates from the low-risk reference on DSS but not from the high-risk group, consistent with intermediate prognosis.

| $K$ | Endpt | Comparison | Event rate $a$ | Event rate $b$ | Log-rank $p$ | $-\log_{10} p$ |
|---|---|---|---|---|---|---|
| 2 | DSS | Cl. 1 vs. Cl. 2 | 42.4% | 11.7% | $6.35\times10^{-10}$ | 9.20 |
| | PFI | Cl. 1 vs. Cl. 2 | 53.5% | 23.1% | $9.02\times10^{-9}$ | 8.05 |
| 3 | DSS | Cl. 1 vs. Cl. 2 | 33.3% | 12.0% | $1.18\times10^{-2}$ | 1.93 |
| | | Cl. 1 vs. Cl. 3 | 33.3% | 42.3% | $1.79\times10^{-1}$ | 0.75 |
| | | Cl. 2 vs. Cl. 3 | 12.0% | 42.3% | $4.18\times10^{-9}$ | 8.38 |
| | PFI | Cl. 1 vs. Cl. 2 | 50.0% | 23.4% | $9.27\times10^{-3}$ | 2.03 |
| | | Cl. 1 vs. Cl. 3 | 50.0% | 51.3% | $4.26\times10^{-1}$ | 0.37 |
| | | Cl. 2 vs. Cl. 3 | 23.4% | 51.3% | $3.49\times10^{-7}$ | 6.46 |
| 4 | DSS | Cl. 1 vs. Cl. 2 | 12.0% | 53.3% | $1.57\times10^{-4}$ | 3.81 |
| | | Cl. 1 vs. Cl. 3 | 12.0% | 16.7% | $6.41\times10^{-1}$ | 0.19 |
| | | Cl. 1 vs. Cl. 4 | 12.0% | 41.3% | $1.87\times10^{-8}$ | 7.73 |
| | | Cl. 2 vs. Cl. 3 | 53.3% | 16.7% | $4.45\times10^{-2}$ | 1.35 |
| | | Cl. 2 vs. Cl. 4 | 53.3% | 41.3% | $9.97\times10^{-1}$ | 0.00 |
| | | Cl. 3 vs. Cl. 4 | 16.7% | 41.3% | $1.91\times10^{-2}$ | 1.72 |
| | PFI | Cl. 1 vs. Cl. 2 | 23.5% | 62.5% | $2.39\times10^{-3}$ | 2.62 |
| | | Cl. 1 vs. Cl. 3 | 23.5% | 32.0% | $5.16\times10^{-1}$ | 0.29 |
| | | Cl. 1 vs. Cl. 4 | 23.5% | 51.3% | $5.32\times10^{-7}$ | 6.27 |
| | | Cl. 2 vs. Cl. 3 | 62.5% | 32.0% | $5.38\times10^{-2}$ | 1.27 |
| | | Cl. 2 vs. Cl. 4 | 62.5% | 51.3% | $9.87\times10^{-1}$ | 0.01 |
| | | Cl. 3 vs. Cl. 4 | 32.0% | 51.3% | $2.55\times10^{-2}$ | 1.59 |

**Table S15** Top mRNA marker genes for KIRC clusters across $K \in \{2, 3, 4\}$, ranked by effect size against the rest of the cohort. Across resolutions, proliferation clusters contain BIRC5, CCNA2, CENPF, RRM2, CDK1, and NDC80; low-risk epithelial/metabolic clusters contain KAT5, EMX2, and ALDH6A1; and mesenchymal/intermediate clusters emerging at $K \geq 3$ contain MARCKSL1, BGN, and JAM3. The additional $K = 4$ Cluster 2 contains SPOCD1 and CDKN3, indicating a chromatin-related signature distinct from the major proliferation cluster.

| $K$ | Cluster | Top marker genes (rank order) |
|---|---|---|
| 2 | 1 ($n = 101$, high-risk, proliferation) | BIRC5, MYBL2, CCNA2, CENPF, CDCA3, RRM2, NDC80, LMNB1, CDK1, SKA3, DEPDC1, C1S |
| | 2 ($n = 213$, low-risk, epithelial) | KAT5, SGSM1, EMX2, MCF2L, PPP1R13B, ALDH6A1, MIR6084, APBB1, CRB3, PLEKHA7 |
| 3 | 1 ($n = 28$, intermediate, mesenchymal) | MARCKSL1, BGN, GBX2, SENCR, C1orf54, ZNF703, RGS3, FBLIM1, PXDN, JAM3, CD248 |
| | 2 ($n = 206$, low-risk, epithelial) | KAT5, EMX2, CRB3, MYL3, AUH, SGSM1, ERBB2, PAIP2B, APBB1, TMEM125 |
| | 3 ($n = 80$, high-risk, proliferation) | PPP1R13B, RRM2, CCNA2, GFOD2, TSPAN7, TMEM88, CENPF, BIRC5, MYBL2, NDC80, CDK1 |
| 4 | 1 ($n = 197$, low-risk, epithelial) | KAT5, EMX2, CRB3, MYL3, AUH, SGSM1, ERBB2, PAIP2B, APBB1, TMEM125, PLEKHA7 |
| | 2 ($n = 16$, high-risk, chromatin) | SPOCD1, CDKN3, NRBP1, USB1, ZYX, CDK2AP1, TEAD3, CAPZA1 |
| | 3 ($n = 25$, intermediate, mesenchymal) | JAM3, TBXA2R, GABRD, ACVRL1, GPR3, CDC42EP2, CCDC184, MARCKSL1, RAMP2, BCL6B |
| | 4 ($n = 76$, high-risk, proliferation) | PPP1R13B, RRM2, CCNA2, GFOD2, TSPAN7, TMEM88, CENPF, BIRC5, MYBL2, INPP5A |

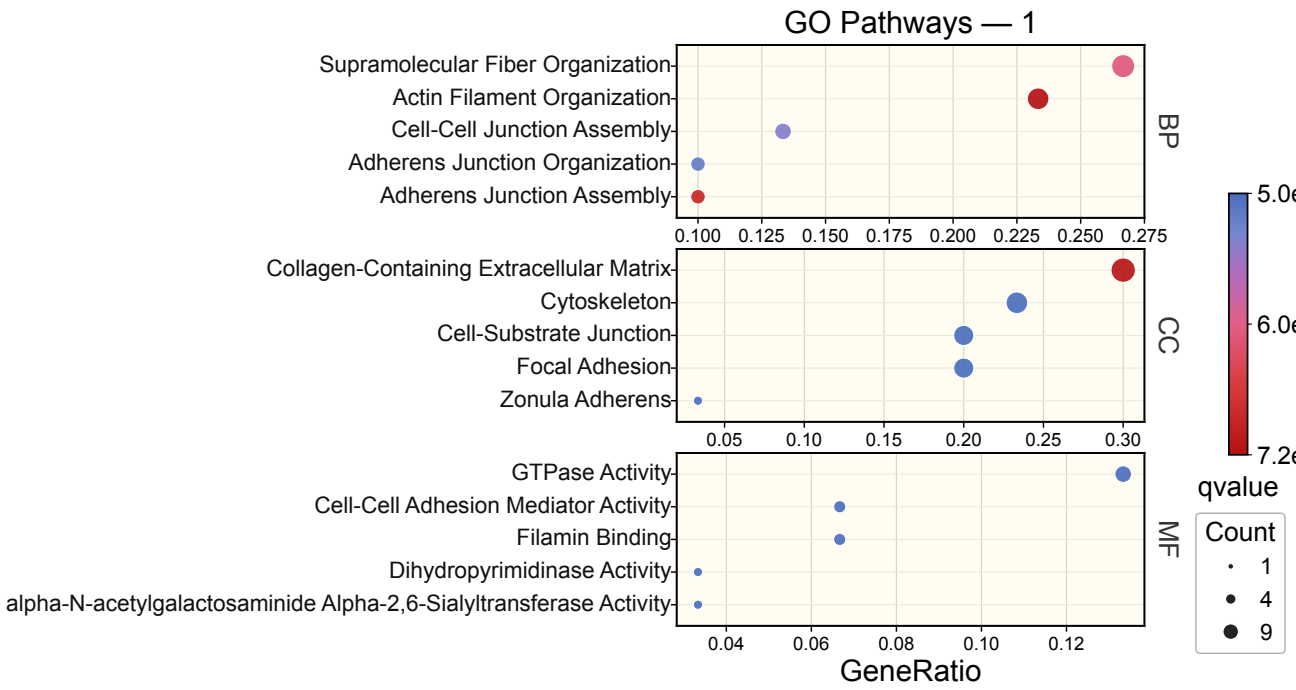


Cluster 1

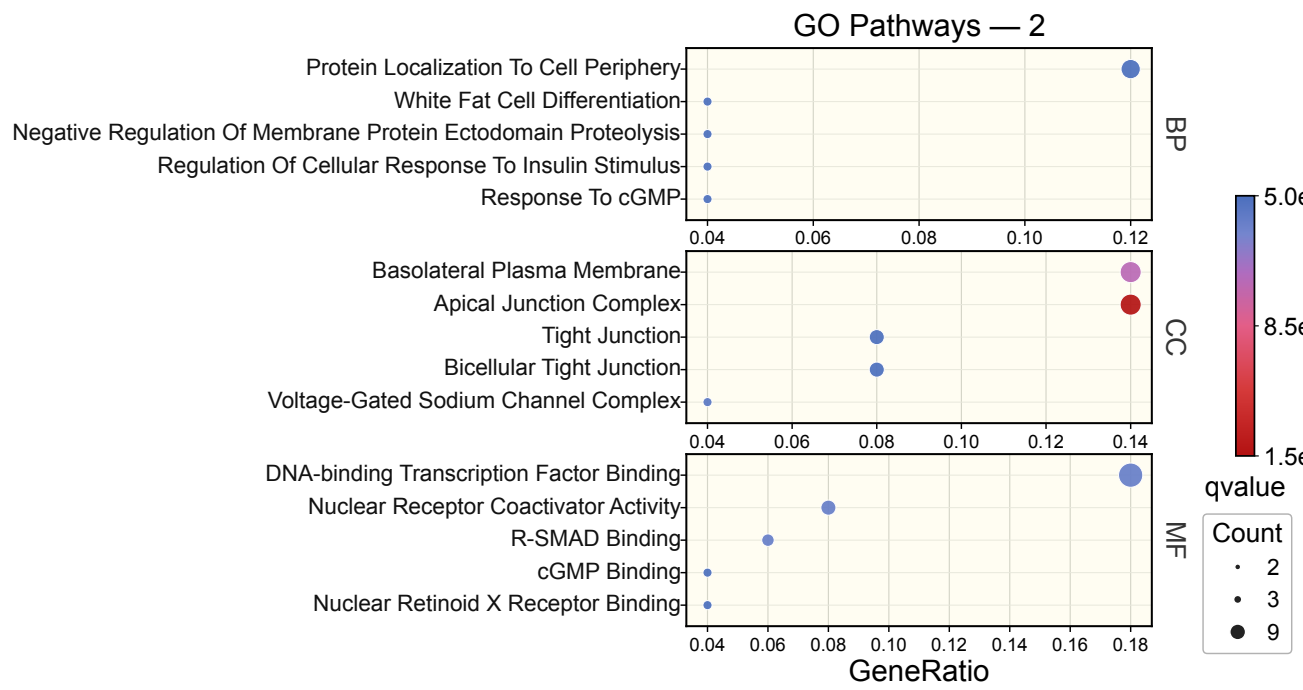


Cluster 2

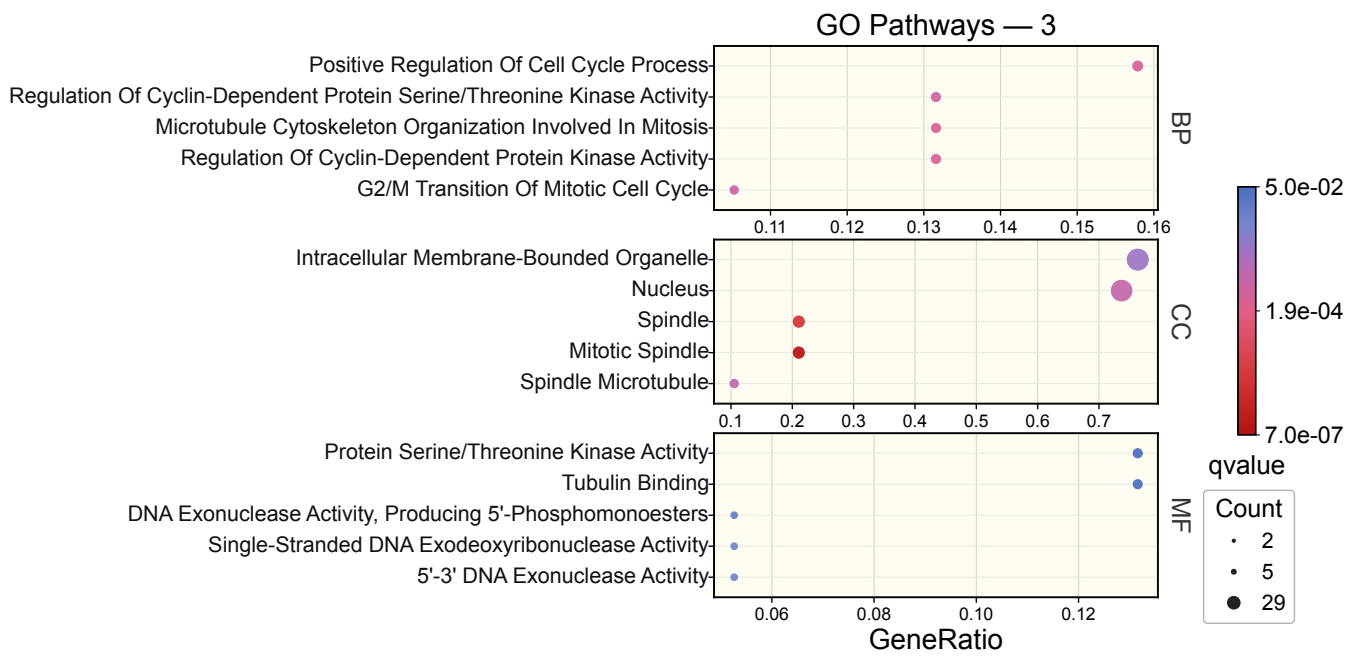


Cluster 3

**Figure S7** Top GO Biological Process enrichment terms for the three KIRC clusters under the selected $K = 3$ resolution.

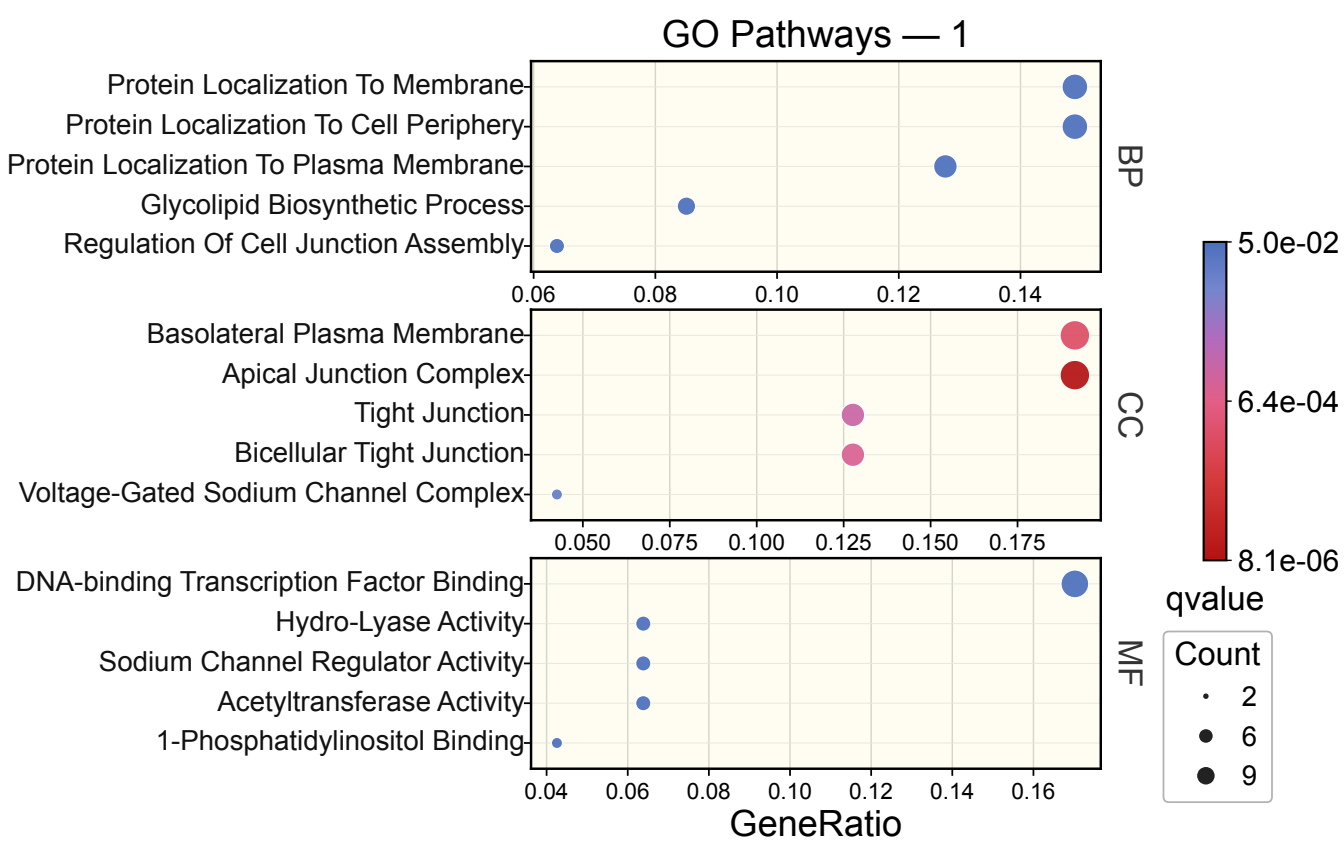


Cluster 1

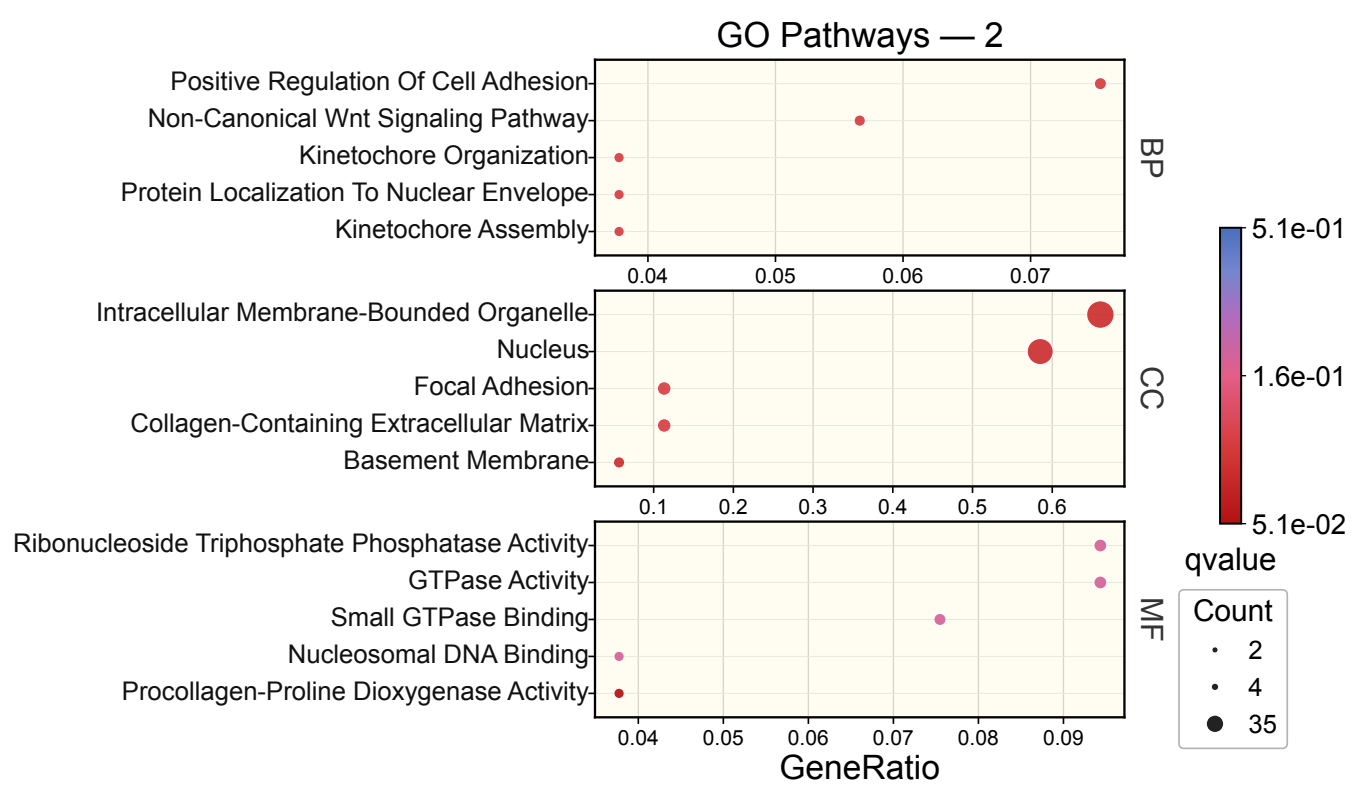


Cluster 2

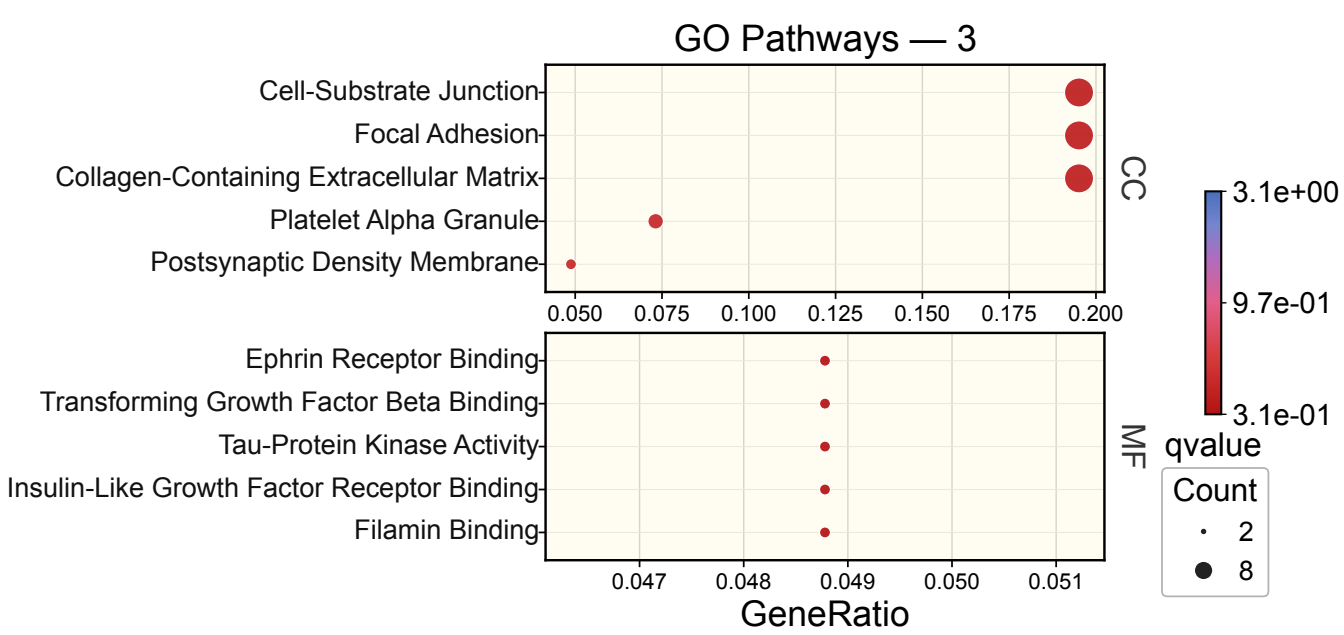


Cluster 3

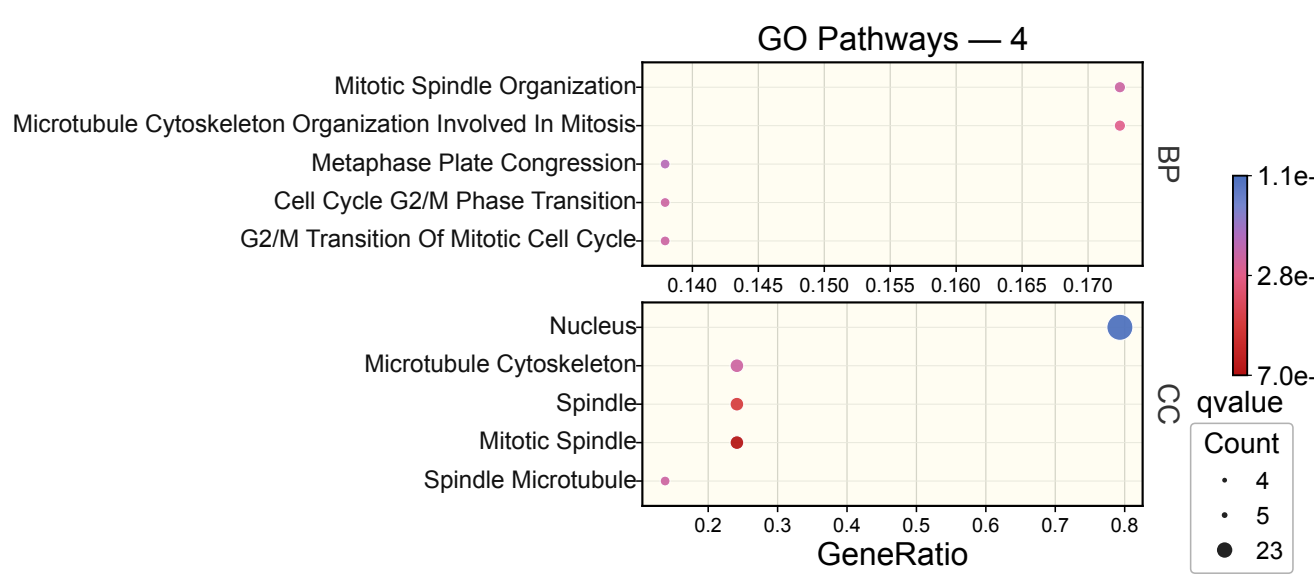


Cluster 4

**Figure S8** Top GO Biological Process enrichment terms for the four KIRC clusters under $K = 4$.
